# Post-COVID impairment of T cell responses to community-acquired pathogens can be modified by activating cellular metabolism

**DOI:** 10.1371/journal.ppat.1013860

**Published:** 2026-07-30

**Authors:** Daniel D. Carroll, Kamacay Cira, Jack Archer, Jason Shapiro, Hardik Shah, Ue-Yu Pen, David Tieri, Lucia Leonor, Neda D. Roofchayee, Samantha S. Yee, Marc Wahab, Igor J. Koralnik, John C. Alverdy, Arjun S. Raman, Lavanya Visvabharathy

**Affiliations:** 1 Department of Surgery, University of Chicago, Chicago, Illinois, United States of America; 2 Department of Surgery, TUM University Hospital, Rechts der Isar, TUM School of Medicine and Health, Technical University of Munich, Munich, Bavaria, Germany; 3 Center for Research Informatics, University of Chicago, Chicago, Illinois, United States of America; 4 Metabolomics Platform, The University of Chicago Medicine Comprehensive Cancer Center, Chicago, Illinois, United States of America; 5 Department of Pathology, University of Chicago, Chicago, Illinois, United States of America; 6 Davee Department of Neurology, Feinberg School of Medicine, Northwestern University, Chicago, Illinois, United States of America; The University of Texas Medical Branch at Galveston, UNITED STATES OF AMERICA

## Abstract

Infection rates involving bacterial and viral pathogens have increased precipitously after the COVID-19 pandemic, though underlying causes remain elusive. Potential causes ranging from increased hospitalizations during the pandemic or greater use of antibiotics have been proposed, but precisely why rates remain high today is unknown. Here, we demonstrate that decreased mitochondrial function in antigen-specific T cells post-COVID may contribute to higher infection susceptibility by metabolically immobilizing T cell responses. Using donor-matched peripheral blood samples from 31 COVID-naïve individuals who subsequently contracted COVID-19, we tracked how influenza A (IAV), *Staphylococcus aureus* (SA), and Varicella-zoster virus (VZV)-stimulated T cell responses were impacted by SARS-CoV-2 infection. Post-COVID CD4 memory T cells exhibited decreased activation- and increased mitochondrial redox-related gene expression. Despite this, mitochondrial flux and reactive oxygen species production were functionally limited in post-COVID antigen-specific T cells after stimulation with IAV, SA, and VZV. Post-COVID plasma was depleted in carnitine and TCA cycle species important for activating fatty acid oxidation, and this correlated with a disordered relationship between memory T cell mobilization of glycolysis, fatty acid metabolism, and oxidative phosphorylation pathways. Metabolic perturbations ultimately resulted in diminished use of catabolic, energy-generating pathways including glycolysis and fatty acid oxidation in antigen-specific T cells. Activating mitochondrial function with metformin and ubiquinol partially rescued the post-COVID decline in T cell catabolism. Collectively, these findings indicate that COVID-19 infection may inhibit T cell metabolism upon exposure to commonly encountered pathogens, which can be partly corrected with common medications that activate mitochondrial metabolism. Our findings may have significant implications for the clinical care of immunologically vulnerable populations in the post-pandemic era.

## Author summary

Infection rates involving bacterial and viral pathogens have increased precipitously after the COVID-19 pandemic, though underlying causes remain elusive. Mitochondrial dysfunction is known to occur post-COVID and may disrupt immune responses. We sought to test this hypothesis using patient-matched pre- and post-COVID immune cells. Here, we demonstrate that decreased mitochondrial function in antigen-specific T cells post-COVID may contribute to higher infection susceptibility by metabolically immobilizing T cell responses. After challenging immune cells with peptides from clinically relevant pathogens (Influenza A-IAV, *Staphylococcus aureus*- SA, and Varicella-zoster virus-VZV), we found global gene expression changes pointing to decreased CD4 memory T cell activation and altered mitochondrial metabolism in post-COVID samples. This correlated with depleted TCA cycle metabolites and carnitine species in post-COVID patient plasma and a disordered activation of metabolic pathways in post-COVID memory T cells. Ultimately, we found lower usage of catabolic pathways in IAV, SA, and VZV antigen-specific memory T cells after COVID-19. We were able to partially rescue metabolic deficiencies after exposure of T cells to drugs that activated mitochondrial metabolism. Overall, our findings may have significant implications for the clinical care of immunologically vulnerable populations in the post-pandemic era.

## Introduction

Infections of all types have increased worldwide after the COVID-19 pandemic. Over the past 3 years (2022–24), there have been outbreaks of viral Mpox [1], co-occurring epidemics of respiratory syncytial virus (RSV), influenza, and COVID-19 [2], as well as increased rates of bacterial infections in hospitalized patients above pre-pandemic levels [3]. Co-occurring epidemics, particularly with pathogens that occupy the same host niche, were previously considered rare. Epidemiological studies on respiratory viral infection cases from 2005-13 found negative temporal correlations between influenza virus, RSV, and other common cold virus rates, demonstrating an ecological “competitive inhibition” when multiple pathogens target the same host niche [4]. However, this pattern has been upended by COVID-19. Frequent observations of co-occurring respiratory virus outbreaks [5] as well as elevated herpes virus reactivation [6] and *Staphylococcus aureus* infections [7] occur during and proximal to a COVID-19 infection. Explanations put forth have ranged from increased hospital usage during the COVID-19 pandemic [8] to greater use of antibiotics [3], but none of these explain why infection rates continue to increase in the present day. We propose an alternate hypothesis: that prior infection with COVID-19 may durably impair T cell responses to other pathogens of clinical relevance by reprogramming mitochondrial metabolism.

Mitochondrial dysfunction is a hallmark of acute and post-acute COVID-19. Patients hospitalized for COVID-19 exhibited unique T cell subsets with downregulated glycolytic proteins which were linked to lymphocyte apoptosis and increased disease severity [9]. Importantly, this phenotype was unique to COVID-19 patients and not observed in those with hepatitis C or those hospitalized for influenza. Increased T cell mitochondrial mass, altered mitochondrial reactive oxygen species (ROS) levels, metabolic quiescence, and disrupted mitochondrial architecture frequently occur during acute COVID-19 [10]. Mitochondrial regulation of T cell metabolism is critical to maintain a balance between effector and memory functions through preferential utilization of glycolysis or oxidative phosphorylation (OXPHOS) [11]. Memory T cells provide a useful example of this balance. At baseline, resting memory T cells have a metabolically quiescent phenotype characterized by lower mitochondrial membrane potential and increased mitochondrial biogenesis, which are desirable features of long-lived cells that protect against reinfection. Upon antigen re-encounter, conversely, memory T cells exploit their higher spare respiratory capacity (SRC) relative to effector cells to provide the ATP necessary through rapid mobilization of glycolysis to differentiate into secondary T effector cells [12]. This process is crucial to mediate their rapid recall ability. Whether and how SARS-CoV-2 infection impacts the critical switch between glycolysis and OXPHOS in T cells to induce broad immunosuppression remains an open question.

Here, we demonstrate that antigen-specific T cells from post-COVID individuals show lower glycolysis usage and disordered mitochondrial metabolism in response to commonly encountered childhood and environmental pathogens. Critically, we studied samples from individuals who were initially COVID-naïve, i.e., they had never been exposed to SARS-CoV-2 at the time of the first blood collection. Using matched samples from the same individuals after infection, we compared T cell responses in pre-COVID and post-COVID peripheral blood mononuclear cells (PBMCs) to IAV, SA, and VZV antigens. Despite unchanged antigen-specific memory T cell percentages, post-COVID memory T cell responses exhibited reduced coordination across glycolysis, fatty-acid synthesis/oxidation, and OXPHOS pathways relative to pre-COVID responses, a pattern associated with higher PD1 expression. This deficit was partly rescued by pharmacological agents that activate mitochondrial complex I and III, demonstrating novel potential avenues for treatment of post-COVID immunosuppression. Overall, we uncover a COVID-related deficit in T cell responses to community-acquired pathogens which may explain the durability of elevated infection rates post-pandemic.

## Results

### Increase in infections and associated mortality rates post-pandemic

**Fig 1:**
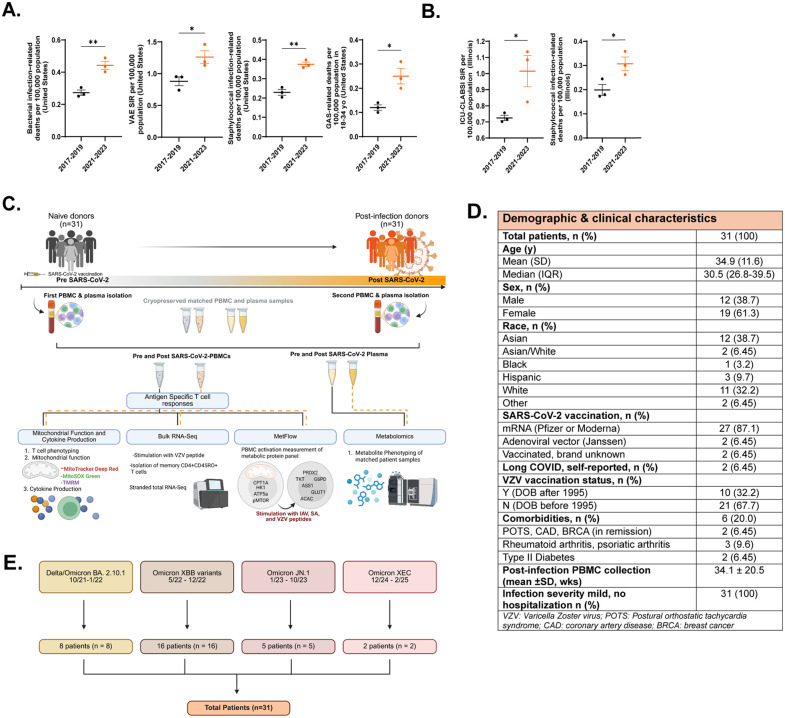
Increased infection rates post-COVID, study design, demographic information. **A.**) Bacterial infection-related deaths, ventilator-associated respiratory infections, *Staphylococcal* infection-related deaths, and Group A Streptococcus (GAS)-related deaths per 100,000 significantly increased in the US during the years after the COVID-19 pandemic. **B.**) Total infection-related deaths, bloodstream infection rates, and *Staphylococcal* infection-related deaths similarly increased in the state of Illinois after the COVID-19 pandemic. **C.**) Study design schematic. **D.**) Study participant demographics including COVID-19 vaccination data. **E.**) Predominant SARS-CoV-2 variant in community circulation at the time of COVID infection for each participant. VAE SIR: ventilator-associated events, standardized infection rate. ICU-CLABSI: intensive care unit-central line-associated bloodstream infections. *p < 0.05, **p < 0.01, ***p < 0.005 by unpaired two-tailed t-test. Error bars show mean ± SEM. Data for A-B gathered from the CDC Wonder database in September 2024. Fig 1C: Created in BioRender. Visvabharathy, L. (2026) https://urldefense.com/v3/__https://BioRender.com/v5ja1hb__;!!MyIu0v6UfBA57LoN!7vUZ9kyk3qCkLXcU3eXdJyQxGv2nwFA5QQZJ-goxoexxrJnzzhDcR6ClzctJR87xgJcEsTzqLlxq35qRbJ_DiMHMYqjUlMz_B6A$. Fig 1E: Created in BioRender. Visvabharathy, L. (2026) https://urldefense.com/v3/__https://BioRender.com/b434mew__;!!MyIu0v6UfBA57LoN!7vUZ9kyk3qCkLXcU3eXdJyQxGv2nwFA5QQZJ-goxoexxrJnzzhDcR6ClzctJR87xgJcEsTzqLlxq35qRbJ_DiMHMYqjUSw4k10E$.

Several studies have reported increased rates of both bacterial and viral infections post-pandemic which prompted us to investigate whether similar trends were evident in publicly available nationwide infection data from the Centers for Disease Control and Prevention (CDC). The CDC WONDER (Wide-ranging Online Data for Epidemiologic Research) database is an online tool to output publicly available data pertaining to morbidity, mortality, and other public health metrics from across the US from 1999-2023 [13]. Data can be parsed by disease type and locale, among other metrics. We limited our search to the 3 years immediately preceding the pandemic (2017–2019) compared to 3 years after the pandemic began (2021–2023), excluding 2020 due to disruptions in data collection which initially arose with COVID-19. Nationwide, death rates associated with bacterial infections significantly increased post-2020, with notable increases in *Staphylococcus* and Group A Streptococcus (GAS) deaths per 100,000 compared to pre-pandemic years (Fig 1A). Ventilator associated infections which frequently occur with respiratory viral infections were similarly increased (Fig 1A). Illinois state level data, where our cohort is based, had similar trends showing a significant upsurge in hospital-acquired bloodstream infections (CLABSI) and *Staphylococcal*-associated deaths (Fig 1B). Though not causal, these data provided a rationale to further explore links between prior SARS-CoV-2 infection and immune suppression against clinically relevant pathogens.

### Mitochondrial oxidative phosphorylation and antigen presentation pathway gene expression in memory T cells is significantly impacted by COVID status

Our group had collected peripheral blood mononuclear cell (PBMC) samples from 31 healthy, COVID-naive donors from 2021-2023 as part of the IRB-STU00212583 study at Northwestern University investigating T cell responses to COVID-19 vaccination. All samples used in the present study were from vaccinated patients. As part of the vaccine study, we found that the primary series of the Pfizer or Moderna mRNA vaccine induced comparable Spike-specific T cell responses in naive individuals compared with healthy COVID convalescents after 6 months (S1 Fig), demonstrating that vaccine-elicited T cell reactivity was similar to that from infection. COVID-naïve status was confirmed by lack of antibody responses to SARS-CoV-2 nucleocapsid protein prior to enrollment (S2A Fig). During the course of the study (2021–25), each participant’s COVID status was confirmed by either PCR- or antigen test for SARS-CoV-2. As we collected samples longitudinally to track vaccine-elicited immunity, we were able to cryobank matched samples from the same individuals pre- and approximately 34 weeks post-COVID infection. One patient contracted COVID twice during the course of the study, and we used samples from all 3 time points in the analysis. Given the increased infection rates and sustained cell and mitochondrial dysfunction post-COVID [14,15], we used these matched samples to formally test the hypothesis that SARS-CoV-2 increases susceptibility to other infections by altering memory T cell metabolic programs (study design in Fig 1C, patient demographics in Fig 1D). COVID variants predominant at the time of infection for each patient are shown in Fig 1E.

**Fig 2:**
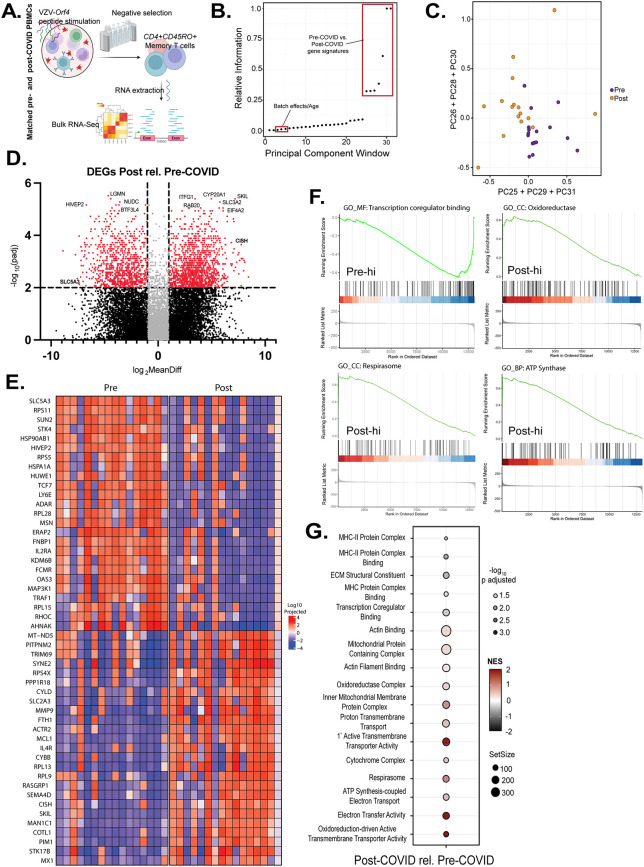
Enhanced mitochondrial bioenergetic pathway gene expression in post-COVID CD4 memory T cells after VZV antigen stimulation. **A.**) Experimental design. **B.**) Statistical information about variance in pre- vs. post-COVID gene signatures are contained in principal components 25-31. **C.**) Isolating principal components 25-31 by using the SCALES method effectively separates samples based on pre- vs. post-COVID gene signatures. **D.**) Differential gene expression by volcano plot in pre- vs. post-COVID CD4 memory T cells. **E.**) Top 25 differentially expressed genes in pre- vs. post-COVID samples. **F.**) Gene set enrichment analysis (GSEA) shows significantly decreased transcription factor binding pathway and elevated ATP synthase, oxidoreductase, and mitochondrial respirasome pathway genes in post-COVID CD4 memory T cells. Pre-hi: pathway expression higher in pre-COVID samples. Post-hi: pathway expression higher in post-COVID samples. **G.**) Significantly altered gene expression pathways enriched in mitochondrial function-related cellular processes in post-COVID relative to pre-COVID CD4 memory T cells. DEGs were calculated based on p_adj_ = 0.05 using a Bonferroni correction for multiple comparisons. Log_2_MeanDiff = mean of gene expression difference, log 2. N = 16 matched pre- and post-COVID samples. Fig 1A: Created in BioRender. Visvabharathy, L. (2026) https://urldefense.com/v3/__https://BioRender.com/s8fna3o__;!!MyIu0v6UfBA57LoN!7vUZ9kyk3qCkLXcU3eXdJyQxGv2nwFA5QQZJ-goxoexxrJnzzhDcR6ClzctJR87xgJcEsTzqLlxq35qRbJ_DiMHMYqjUifz9Mq4$.

To determine the impact of COVID-19 on T cell memory, we initially investigated Varicella-zoster virus (VZV) responses. We chose VZV for the following reasons. First, all individuals would have been exposed either from natural infection or vaccination in childhood. In addition, VZV-specific recall responses remain stable over periods of 24 months or more and persist at high levels into adulthood, making it unlikely for VZV responses to change across the sampling interval [16]. Reports have also credibly shown that increased VZV reactivation is found in COVID-19 survivors [17]. Pre-COVID samples had a 93% VZV antibody positivity rate (S2B Fig), indicating that the majority of study participants had humoral immune memory to VZV. This established a uniform baseline of exposure against which to interrogate post-COVID qualitative changes in T memory cell function at the population level. Sixteen subjects were randomly selected from the original cohort of thirty-one, and their PBMCs were stimulated with a VZV Orf4 peptide array spanning the entire protein (antigen descriptions in S2C Fig). We then purified CD4 + CD45RO+ memory T cells by negative selection (purity in S3 Fig) before isolating RNA. Further analysis was conducted with bulk RNA sequencing, which provided us with a transcriptional signature of pre- and post-COVID T cell gene expression (Fig 2A).

To reveal differences between these two cell populations, we turned to dimensionality reduction approaches typically used in analyzing high-content biological data [18]. We first performed Principal Components Analysis (PCA), defining a set of 31 principal components (PCs). In general, PCA focuses signals of statistical variation amongst the so-called ‘top-modes’ of variance—modes that harbor the most amount of variation within the data. Analysis of the top PCs in our data illustrated a statistically significant, non-random relationship with batch effect and age of the patient from which the sample was collected (S4A Fig). Further analysis using non-linear dimension-reduction methods—UMAP and t-SNE—recapitulated the same finding (S4B Fig). These results suggested that the most global signals of variation in our data were unrelated to pre- versus post-COVID signatures of T cell modification.

A parsimonious explanation of our findings would suggest that there may not exist a transcriptional signature separating pre- from post-COVID T cells. However, recently created statistical approaches, developed within the field of bacterial genomics and phylogenetic analysis, have suggested that relevant biological signal may actually reside in PCs that harbor substantially less variation than the top PCs. This is because the spectrum of principal components encodes a tree of relatedness amongst systems, where biological information captured within the lowest PCs is statistically nested within the information captured by the highest, most dominant PCs.

With this nested data structure in mind, we next analyzed our data using a framework called SCALES (Spectral Correlation Analysis of Layered Evolutionary Signals)—a statistical approach that performs a data-driven search across all PCs to identify regions of the PC spectrum most associated with the desired phenotype [18]. (Fig 2B). Using SCALES, we were able to identify that while the first PCs contained mostly information related to various batch effects, information specific to phenotypic differences was isolated at later PCs (primarily PC25 - PC31), where the batch effects are already minimized. The benefit of the SCALES approach is it enabled us to explore differential expression by creating projected counts based only on these relevant PCs. This is shown in Fig 2C, where a combination of 6 later PCs cleanly separates Pre- and Post-COVID samples. As a result, there is no need for additional batch correction when comparing expression based only on this subset of PCs.

Having identified PCs encoding information relaying a “COVID effect”, we revealed distinct transcriptional signatures separating pre- and post-COVID states (Fig 2B,2C). Differential expression analysis identified extensive transcriptional remodeling, with 2048 differentially expressed genes (DEGs) between pre- and post-COVID samples (p_adj_ < 0.05, log_2_MeanDiff >1; Fig 2D), where log_2_MeanDiff denotes the log_2_ difference between the mean projected expression in post- versus pre-COVID samples along the selected principal components. Transcripts most strongly downregulated post-COVID included solute carrier family 5 member 3 (*SLC5A3*), transcription factor 7 (*TCF7*), lymphocyte antigen 6 family member E (*LY6E*), endoplasmic reticulum aminopeptidase 2 (*ERAP2*), and interleukin-2 receptor subunit alpha (*IL2RA*), among others. These genes are involved in inositol import and calcium mobilization for T cell activation (*SLC5A3*), memory T-cell differentiation [19] (*TCF7*), coronavirus entry restriction factor [20] (*LY6E*), and cytokine-driven proliferation (*IL2RA*). In contrast, post-COVID samples were enriched for MX dynamin-like GTPase 1 which may promote viral persistence [21] (*MX1*), cytokine-inducible SH2-containing protein which suppresses T cell activation [22] (*CISH*), and mitochondrially encoded NADH dehydrogenase 5 (*MT-ND5*), among others (Fig 2E). Importantly, pathway analysis found significant downregulations in MHC-II complex binding and transcription factor co-regulation, but upregulated mitochondrial OXPHOS and ATP synthesis pathways in post-COVID T cells (Fig 2F, 2G). Together, these results demonstrate that SARS-CoV-2 infection is accompanied by durable transcriptional reprogramming of CD4 ⁺ memory T cells after exposure to VZV antigens, possibly through bystander effects, defined by enrichment of genes pertinent to mitochondrial metabolism. This is consistent with a metabolic shift that may contribute to the functional impairments of CD4 ⁺ memory T cells at the population level within this cohort.

### Bimodal phenotype of mitochondrial function in antigen-specific memory T cells post-COVID

Because post-COVID memory T cells showed enrichment of mitochondrial-metabolic pathway genes, we inferred that these transcriptional shifts indicate functional metabolic changes impacting antigen-specific memory responses. To test this, we expanded our analyses beyond VZV to include Influenza A virus (IAV) and *Staphylococcus aureus* (SA) antigens representing infections of broader clinical relevance (antigen descriptions in S1C Fig). All participants showed prior exposure to these pathogens, confirmed serologically (S1D Fig).

**Fig 3:**
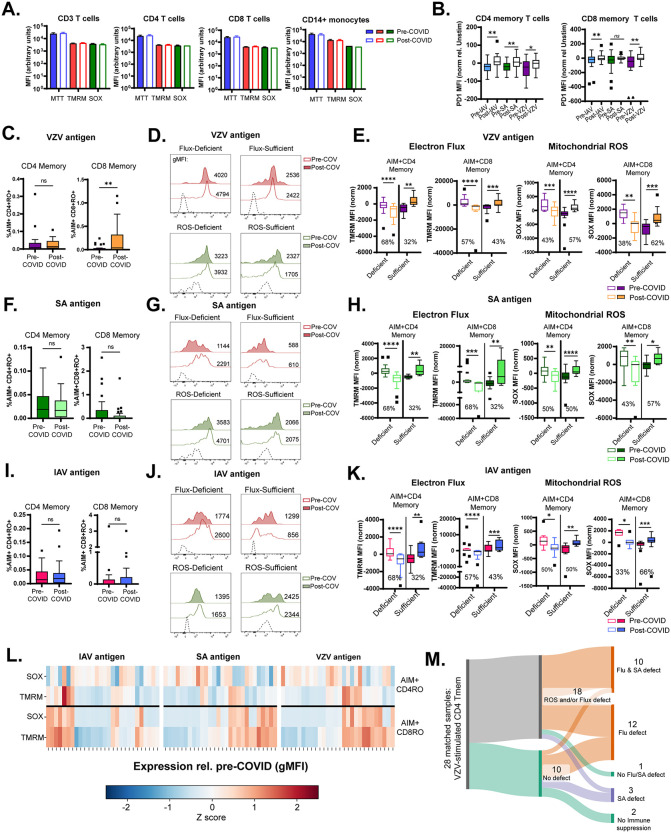
Diminished mitochondrial function in antigen-specific post-COVID memory T cells. **A.**) Baseline levels of mitochondrial mass (MTT), mitochondrial electron flux (TMRM), and mitochondrial reactive oxygen species production (SOX) in immune cell subsets are unchanged in unstimulated pre- and post-COVID immune cells. **B.**) Elevated PD1 expression in antigen-stimulated memory T cells post-COVID. **C.**) VZV antigen-specific (AIM+) CD4 memory T cell numbers are unchanged after COVID, while AIM + CD8 memory T cell numbers are elevated. **D-E.**) Mitochondrial ROS and mitochondrial flux post-COVID in VZV antigen-specific (AIM+) memory T cells. A majority of study subjects produced less mitochondrial ROS and displayed lower electron flux post-COVID. **F.**) Levels of SA antigen-specific memory T cells are unchanged post-COVID. **G-H.**) Deficiency in electron flux and mitochondrial ROS production in SA antigen-specific memory T cells in a majority of subjects. **I.**) Unchanged percentages of IAV antigen-specific memory T cells post-COVID. **J-K.**) Similar to VZV and SA, mitochondrial ROS and mitochondrial flux deficiency is evident post-COVID in IAV antigen-specific memory T cells from a majority of samples. **L.**) Heatmap of mitochondrial ROS and mitochondrial flux expression across all samples. **M.**) Flow diagram showing that 92.8% of subjects had ROS and/or flux deficiency to at least one set of antigens. Graphs show mean ±SEM. *p < 0.05, **p < 0.01, ***p < 0.005, ****p < 0.0001 by two-tailed paired Student’s t test or Wilcoxon test depending on normality of the data from n = 28 individuals, matched pre- and post-COVID samples. C, F, I: representative data from 1/28 individuals; dashed histogram represents FMO. E, H, K: gMFI data were normalized by subtracting Ag-stimulated conditions from the unstimulated baseline condition for each sample, sometimes resulting in negative numbers indicating decreased gMFI compared to baseline. E, H, K: percentages under bar plots represent fraction of patients either “deficient” or “sufficient” for mitochondrial function (total n = 28).

We first assessed mitochondrial activity at baseline using functional dyes: Mitotracker (MTT) to detect active mitochondrial mass, tetramethylrhodamine methyl ester (TMRM) to assess mitochondrial membrane potential and electron flux, and MitoSOX (SOX) to detect mitochondrial reactive oxygen species (ROS) production. Baseline values were comparable across unstimulated CD3, CD4, CD8 T cells and CD14 monocytes pre- and post-COVID (Fig 3A; gating in S5 Fig), though we did find increased PD1 expression in antigen-stimulated post-COVID CD4 and CD8 memory T cells suggestive of T cell exhaustion (Fig 3B). The overall frequencies of antigen-specific activation-induced marker (AIM+ as defined in [23]; gating in. S6 Fig) CD4⁺ and CD8 ⁺ memory T cells recognizing VZV, SA, or IAV peptides were preserved, apart from a modest increase in VZV-specific AIM + CD8 ⁺ memory T cells post-COVID (Fig 3C, F, I). These results indicate that SARS-CoV-2 infection does not substantially alter the pool size of antigen-specific memory T cells but may increase T cell exhaustion in the memory compartment.

Functional analyses then revealed post-COVID impairments in mitochondrial responses exhibited a bimodal distribution. VZV-specific CD4⁺ and CD8 ⁺ memory T cells from more than half of the study participants had significantly lower mitochondrial flux and ROS production post-COVID compared to paired pre-COVID samples (Fig 3D, 3E). Comparable patterns were observed following stimulation with SA (Fig 3G, 3H) and IAV (Fig 3J, 3K). No significant differences were found in mitochondrial mass when parsed by pre- vs. post-COVID status, with the exception of SA-stimulated CD4 memory T cells (S7 Fig). The population distribution of TMRM and SOX expression in memory T cells is summarized as a heatmap in Fig 3L. Across all antigen stimulations, 33–79% of individuals displayed post-COVID deficits in at least one mitochondrial function parameter. A participant-level flow diagram confirmed the breadth of these defects: 93% of subjects exhibited impaired mitochondrial function in antigen-specific CD4 ⁺ memory T cells for at least one pathogen, and most showed deficits across multiple pathogens (Fig 3M). Together, these findings demonstrate that although antigen-specific memory frequencies remain stable, SARS-CoV-2 infection is associated with broad, pathogen-independent impairments in mitochondrial bioenergetics underpinning T cell recall responses.

### Metabolic alterations in post-COVID memory T cells

**Fig 4:**
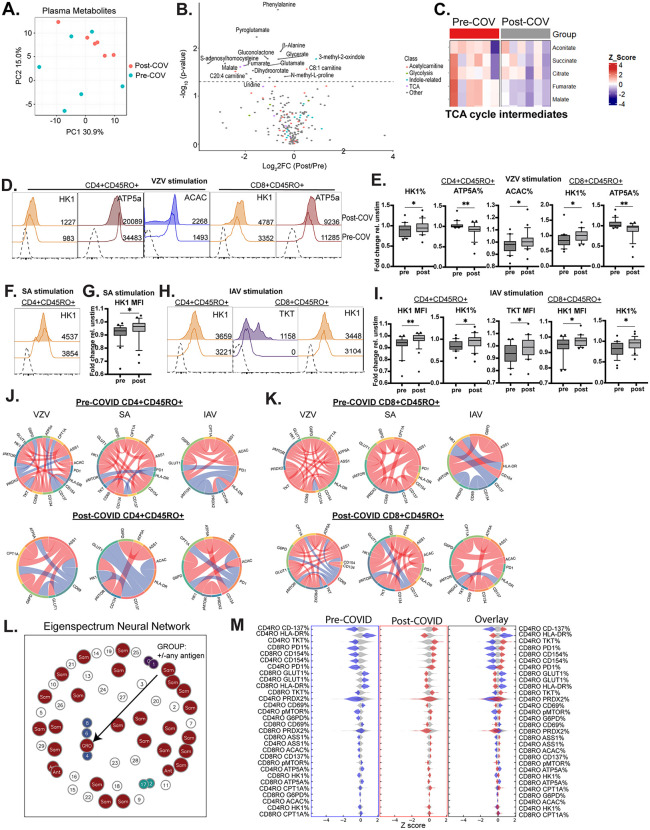
Metabolic pathways are disordered in post-COVID memory T cells and correlated with increased T cell exhaustion. **A.**) PCA plot differentiating pre- and post-COVID plasma samples by metabolite abundance. **B.**) Volcano plot showing significant decreases in circulating carnitine species and TCA cycle intermediates in post-COVID plasma samples. **C.**) Relative abundance of specific TCA cycle intermediates in pre- and post-COVID plasma*.* **D-E.**) Post-COVID elevations in HK1 (glycolysis) and ACAC (fatty acid synthesis) and decreased expression of ATP5a (OXPHOS) in memory T cells after VZV antigen stimulation. **F-G.**) Higher HK1 expression in SA antigen-stimulated CD4 memory T cells post-COVID. **H-I**). Elevated HK1 expression and TKT expression (both glycolysis enzymes) in IAV-stimulated memory T cells after COVID. **J-K.**) Connectivity between metabolic pathways (% enzyme or activation marker positive memory T cells) is lost post-COVID in both CD4 (J) and CD8 (K) memory T cells. The loss of connectivity is more pronounced in CD4 memory T cells. Blue arcs: positive correlation; red arcs: negative correlation. **L.**) Eigenspectrum neural network analysis of statistical connections within CD4 and CD8 memory T cells finds a group-specific pre- vs. post-COVID signature. **M.**) Feature distribution map differentiates pre- vs. post-COVID memory T cell phenotypes. Blue: post-COVID; red: pre-COVID. The top 13 features explain the majority of statistical variation between pre- and post-COVID groups (overlay). Filled histograms: post-COVID; open histograms: pre-COVID. Graphs show mean ±SEM. *p < 0.05, **p < 0.01 by two-tailed paired Student’s t test in n = 24 samples. J, K: chord diagrams of Pearson correlations in % of memory T cells positive for the specified marker. Unit of analysis: CD4 + RO+ or CD8 + RO+ cells from n = 24 matched pre- and post-COVID patient samples. Significance thresholds were set at |R| > 0.5. L, M: data from D-K used to generate feature maps using Boltzmann Brain analysis. Feature maps show clear segregation of biomarkers differentiating pre-COVID (CD137^lo^, HLA-DR^hi^, PD1^lo^, GLUT1^hi^) from post-COVID (CD137^hi^, HLA-DR^lo^, PD1^hi^, GLUT1^lo^) T cell states.

Given post-COVIDdisruption of mitochondrial function in antigen-specific memory T cells, we probed whether metabolite profiles were altered in post-COVID plasma compared to their pre-COVID controls. We conducted polar metabolite profiling on six paired plasma samples and found that PCA effectively separated pre- and post-COVID metabolite profiles (Fig 4A). Further analysis revealed depleted carnitine species, malate, and fumarate compared to pre-infection controls (Fig 4B) suggesting infection-associated deficiencies in the TCA cycle and fatty acid oxidation occurs as in chronic viral infections [24] or long COVID [25]. The relative abundance of specific TCA cycle intermediates in pre- and post-COVID samples is shown in Fig 4C.

Plasma metabolite alterations reflected global metabolic changes, leading us to define the usage of metabolic pathways in activated T cells. We employed the Met-Flow technique [26] which uses high-dimensional spectral flow cytometry to detect rate-limiting enzymes involved in glycolysis, oxidative phosphorylation, and fatty acid oxidation/synthesis. We can thus characterize and quantify metabolic alterations associated with T cell survival, differentiation, activation, and function at the single cell level, allowing for subset-specific data collection. We focused on the following enzyme targets: GLUT1, HK1, G6PD, TKT, PRDX2, ASS1, ACAC, IDH2, CPT1A, and ATP5a (diagram and enzyme functions: S8 Fig) involved in glycolysis, fatty acid metabolism, arginine metabolism, and OXPHOS.

At baseline, there were few differences in memory T cell expression of key metabolic enzymes post-COVID, apart from elevated HK1 levels in CD8 memory cells (S9 Fig). However, VZV antigen stimulation led to higher HK1 and ACAC expression but lower ATP5a expression in post-COVID memory T cells compared to matched pre-COVID controls (Fig 4D-4E; all data show fold change relative to unstimulated samples). Elevated HK1 and TKT expression were also found after SA (Fig 4F-4G) and IAV antigen stimulation (Fig 4H-4I), demonstrating that usage of glycolysis pathways was elevated but OXPHOS was lower at the memory T cell population level after COVID. Though statistically significant, the low fold change in some enzyme levels makes it difficult to assess biological significance without further experimentation. Post-COVID memory T cells displayed also markedly diminished connectivity between metabolic enzyme expression and key activation markers such as CD69, HLA-DR, and CD137 compared to matched pre-COVID controls (Fig 4J, 4K).

To better define data features underlying the lack of metabolic connectivity in memory T cells, we sought to create a statistical embedding that differentiated pre- versus post-COVID pathways. To do this, we employed an analysis program called the Boltzmann Brain—a program that constructs embeddings from the eigenspectrum of data. While detailed in Methods, briefly this program conducts a data-driven search for embeddings that are maximally information-rich with respect to a user-defined phenotype of interest. The embeddings can then be queried to discover features that are a statistically inferred ‘biomarker’ of phenotype. In our case, Boltzmann analysis revealed a unique relationship between “Group” (pre- or post-COVID) and “Antigen” (VZV/SA/IAV stimulation) when evaluating memory T cell expression of metabolic and activation markers (Fig 4L), the same underlying data that we used to generate Figs 4D-4K. We analyzed the feature distribution defining antigen stimulation of memory T cells and found distinct biomarker signatures differentiating samples by pre- vs. post-COVID status (Fig 4M). The top features responsible for the largest differences showed elevated HLA-DR and GLUT1 in pre-COVID samples but enriched CD137, TKT, and PD1 expression in post-COVID samples. These data demonstrate that post-COVID memory responses are characterized by diminished connectivity between metabolic pathways but higher glycolysis usage and expression of the inhibitory marker PD1 after antigen stimulation at the cell population level.

Finally, we assessed cytokine production from pre- and post-COVID T cells to link metabolic changes to functional incapacitation. Samples were obtained from nine individuals before and after their first COVID infection or after multiple infections due to naïve sample availability constraints (demographics in S10A Fig). We found that post-COVID T cells had an expanded FoxP3^+^ Treg population, but also secreted higher IFN-γ and IL-6 in the absence of antigen stimulation after 72h in culture (S10B, S10C Fig), demonstrating a disordered relationship between immune suppression and activation at baseline. However, activation of T cells with αCD3/CD28 induced significantly lower IFN-γ production from post-COVID PBMCs at 72h (S10D Fig) which effectively demonstrated functional deficits mirroring metabolic deficits. TCF1 + PD1 + stem-like CD8 memory T cells were highly elevated at baseline in post-COVID samples (S10E Fig), a population known to expand during chronic viral infections [27] that act as precursors of exhausted T cells (T_pex_) [28]. In contrast, T cell activation led to an expansion of stem-like TCF1 + CD4 memory T cells important for generation of T cell memory in pre-COVID but not post-COVID samples (S10F Fig). These data confirm that post-COVID immune cells have a lower cytokine-producing capacity compared to matched pre-COVID controls while also exhibiting multiple markers of immune suppression at baseline (FoxP3 + Treg and PD1 + TCF1 + T_pex_).

### Antigen-specific memory T cells are deficient in glycolysis/fatty acid oxidation usage post-COVID, which is partly reversed by mitochondrial activation

**Fig 5:**
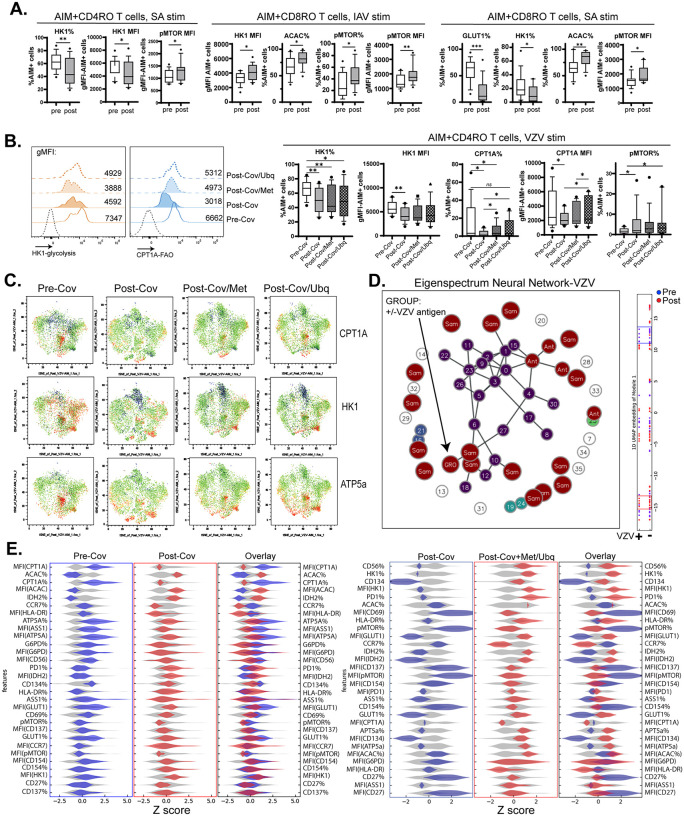
Post-COVID antigen-specific memory T cells exhibit lower usage of glycolysis and fatty acid oxidation, which is partly reversed by activating cellular metabolism. **A.**) Decreased glycolysis (GLUT1, HK1) but elevated fatty acid synthesis (ACAC) and phosphor-mTOR in SA and IAV antigen-specific memory T cells post-COVID. **B.**) Deficits in glycolysis and fatty acid oxidation in VZV antigen-specific CD4 memory T cells can be partly rescued by modulation of mitochondrial complex I (Met, dotted filled histogram) or complex III (Ubq, dotted open histogram). **C.**) Expression of CPT1A, HK1, and ATP5a in AIM + VZV-specific CD4 memory T cells is partly rescued by exposure to Met or Ubq. UMAP data from n = 16 concatenated samples. **D.**) Eigenspectrum neural network of VZV antigen-specific CD4 T cells after discovery of a pre- vs. post-COVID (“Group”) MetFlow signature. Bar to the right represents 1D UMAP representation of data where blue dots are pre-COVID samples and red dots are post-COVID samples. Red and blue windows represent areas with the greatest feature divergence between pre- and post-COVID VZV-specific CD4 memory T cells. Feature maps in 5E were derived from information contained within these windows. **E.**) *Left:* Feature distribution map of pre- vs. post-COVID VZV-specific CD4 memory T cells showing higher glycolysis (GLUT1, HK1), lower immunosuppression (pMTOR), and higher fatty acid oxidation (CPT1A) in pre-COVID samples relative to post-COVID samples. *Right:* Feature distribution map of post-COVID VZV-specific T cells treated with Met or Ubq shows higher expression of glycolytic enzymes (HK1), activation markers (CD134, HLA-DR), and lower pMTOR expression compared to untreated T cells. Plasma samples in Fig 4A-4C, n = 6. Graphs show mean ±SEM. *p < 0.05, **p < 0.01 by two-tailed paired Wilcoxon test in n = 16 samples for Fig 4D-4M.

Though memory T cell responses at the *population* level showed increased glycolysis usage with elevated HK1 expression (Fig 4), this did not necessarily reflect the behavior of *antigen-specific* memory T cells. To address this, we assessed the response of AIM + CD4 and CD8 memory T cells to IAV, SA, and VZV stimulation. In contrast to the overall cell population, AIM+ memory T cells displayed significantly *lower* HK1 and CPT1A expression (representing glycolysis and FAO metabolic pathways) but higher ACAC and pMTOR expression (representing FAS and cell proliferation pathways) indicating mobilization of anabolic pathways, but diminished use of the catabolic glycolysis and fatty acid oxidation after antigen stimulation (Fig 5A-5B). These results corroborated our findings showingdecreased carnitine metabolites important for fatty acid oxidation in post-COVID plasma (Fig 4B).

The switch from OXPHOS to glycolysis is essential for memory T cells to quickly respond to pathogen re-encounter. Similarly, memory T cells rely on fatty acid oxidation to activate effector programs [29]. Given that post-COVID antigen-specific memory T cells were deficient in expression of the glycolytic and FAO enzymes, we determined whether pharmacological modulation of mitochondrial function could rescue post-COVID deficits in cellular metabolism. We focused on CD4 memory responses to VZV because of transcriptional (Fig 2) and mitochondrial function (Fig 3) signatures were able to discriminate between pre- and post-COVID groups. Metformin and ubiquinol are two modulators of mitochondrial function with distinct mechanisms. Metformin is a common anti-diabetic drug that can lower blood glucose levels by promoting glycolysis/FAO and decreasing mitochondrial complex I activation [30]. It has also been shown to promote memory T cell responses against infections and tumors [31,32]. Ubiquinol (also known as coenzyme Q10) is a potent antioxidant that limits inflammation and activates mitochondrial complex III which can promote T cell memory generation to infections and cancer [33].

Incubation of PBMCs with 10µM metformin or 5µM ubiquinol had a demonstrable effect in enhancing CPT1A but not HK1 expression without changing cell viability (S11 Fig) in VZV-specific CD4 memory T cells (Fig 5B), though not to pre-COVID levels. VZV-specific T cells increased CPT1A, and ATP5a expression after drug treatment, with ubiquinol more potent than metformin in promoting expression (Fig 5C). After analysis with the Boltzmann Brain program (Fig 5D), we identified several data features that distinguished pre- and post-COVID VZV-specific memory T cells and unique signatures of post-COVID T cells after drug treatment. Pre-COVID VZV-specific T cells displayed elevated glucose import (higher GLUT1 expression), costimulation/activation (CD134 expression), and lower fatty acid synthesis (ACAC expression) in addition to elevated glycolysis/FAO (HK1/CPT1A expression; Fig 5E, left panel). Metformin or ubiquinol treatment was able to partially restore the post-COVID deficits in HK1 expression, CD134 expression, and GLUT1 expression (Fig 5E, right panel), demonstrating that common pharmacological modulators of mitochondrial metabolism may help correct post-COVID deficits in T cell memory responses.

**Fig 6:**
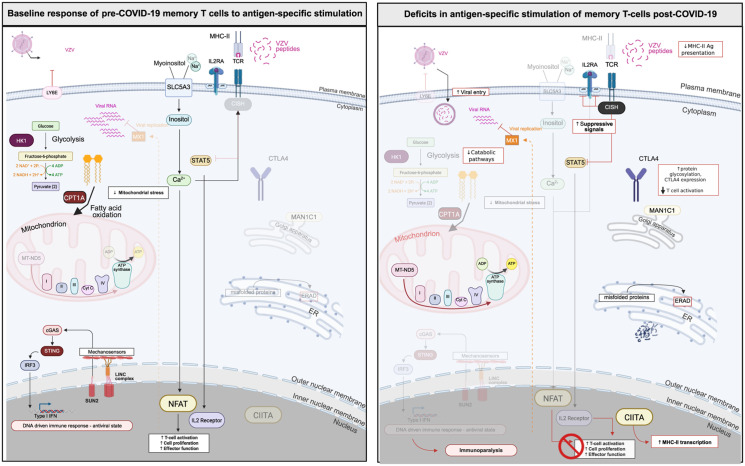
Model of post-COVID modifications of antigen-specific T cell activation driven by metabolic reprogramming. Created in BioRender. Carroll, D. (2026) https://urldefense.com/v3/__https://BioRender.com/ukthmh3__;!!MyIu0v6UfBA57LoN!7vUZ9kyk3qCkLXcU3eXdJyQxGv2nwFA5QQZJ-goxoexxrJnzzhDcR6ClzctJR87xgJcEsTzqLlxq35qRbJ_DiMHMYqjUaTFkUX0$.

Taken together, we have shown that even mild SARS-CoV-2 infection may have lasting effects on T cell memory responses to other pathogens by limiting catabolic pathways such as glycolysis and fatty acid oxidation (Fig 6, model). This may contribute to increased infection rates in community and hospital settings post-pandemic and is relevant to the clinical management of infections in the post-pandemic era.

## Discussion

Post-pandemic, the US and the world at large have entered a “new normal” where overall morbidity and mortality rates have increased. Recent data from the UK revealed an excess death rate of 7.2% in 2022 and 8.6% in 2023 above expectations, despite the peak of the COVID-19 pandemic having passed [34]. Much of the increase in mortality can be attributed to higher rates of infections [35], which raises the following question: *does SARS-CoV-2 infection weaken host immune responses to other clinically relevant infections?* This is the fundamental issue concerning this study.

Effective prevention and treatment of infectious diseases represent one of the seminal advances of medical science. The COVID-19 pandemic, however, reminded us that infections remain deadly and have health consequences beyond the acute infection stage. Here, we demonstrate that non-COVID infection rates in the US have increased in the years immediately following the introduction of COVID-19, which may be linked to immunometabolic dysfunction in the T cell compartment. We found that post-COVID CD4 memory T cells have a unique transcriptional signature enriched in mitochondrial respiration pathways but deficient in T cell activation/proliferation pathways. Further, a majority of individuals exhibited post-COVID reductions of mitochondrial flux and ROS in antigen-specific memory T cells, deficiencies in TCA cycle metabolites in the plasma, diminished connectivity of metabolic pathways (glycolysis, FAO, OXPHOS) in memory T cells, and an inability of antigen-specific T cells to mobilize catabolic pathways (glycolysis, FAO) after antigen stimulation (model in Fig 6). These deficits were partially rescued by exposure to mitochondrial modulator drugs which may represent novel methods for immune reconstitution after COVID-19.

Multiple surveillance efforts have documented a sustained rise in infection rates across bacterial and viral pathogens in the post-pandemic period [36,37]. Importantly, this may be a unique consequence of COVID-19: patients with a positive COVID test between 2021 and 2023 had a significantly higher likelihood to contract bacterial bloodstream and urinary tract infections as well as herpes and respiratory viral infections compared with those seen for influenza A infection [36]. Mechanistically, T cell exhaustion and mitochondrial dysfunction have also been implicated in the pathogenesis of long COVID, where oxidative stress, dysregulated interferon signaling, and persistent immune activation promote durable immunosuppression [12,13]. These converging lines of evidence suggest that SARS-CoV-2–induced dysregulation of adaptive immunity may not only compromise memory responses but also increase susceptibility to future outbreaks, including those of vaccine-preventable diseases.

The recall capacity of memory T cells is highly dependent on mitochondrial agility-i.e., the ability to switch between slow but efficient ATP generation with OXPHOS to quick but inefficient ATP generation with glycolysis and fatty acid oxidation [38]. We found a unique post-COVID transcriptional signature in antigen-stimulated memory T cells with upregulation of immunosuppressive pathways and genes linked to mitochondrial respiration. The immunosuppressive genes *MAN1C1* which increases CTLA4 expression [39] and *CISH*, an active suppressor of TCR signaling [22], were also upregulated post-COVID while the myo-inositol transporter gene *SLC5A3* linked to NF-κB activation and T cell stimulatory gene *IL2Rb* were downregulated after infection. Importantly, activation of T cells through TCR engagement resulted in lower expression of TCF1 (S10F Fig), the protein encoded by the *TCF7* gene which was significantly decreased in post-COVID CD4 memory T cells. TCF1 is a critical mediator of memory T cell “stemness”, i.e., the ability to proliferate after antigen stimulation [40]. Our data suggest this ability is impaired after COVID-19. The *MX1* gene also remained upregulated in post-COVID samples despite being a marker of early and active SARS-CoV-2 infection which declined with viral clearance in patients enrolled in a human challenge study [41]. Given the considerable evidence that SARS-CoV-2 can persist in multiple tissues for months to years after infection [42], it is possible that *MX1* upregulation suggests viral persistence and plays a role in post-COVID T cell dysfunction, though we were unable to detect SARS-CoV-2 in PBMCs from our post-COVID samples (S12 Fig). Perhaps most importantly, mitochondrial oxidoreductase and respirasome genes were significantly upregulated after infection, suggesting that mitochondrial metabolism is disrupted in antigen-stimulated memory T cells after COVID-19 exposure. Mitochondrial dysfunction has routinely been identified within immune cells of long COVID patients [43] and in COVID convalescents more broadly [44], but we are the first to our knowledge to discover underlying mitochondrial dysfunction in antigen-specific memory T cells from patients *without* long COVID at 3–9 months after infection.

Given that plasma metabolite profiling demonstrated post-COVID depletion of critical carnitine and TCA cycle metabolites (Fig 4A-4C), we reasoned that decreased mobilization of energy-generating pathways after T cell activation was responsible. Importantly, we found that glycolysis-related enzymes were upregulated in memory T cells at the population level but downregulated in antigen-specific memory T cells post-COVID. This validates our initial hypothesis that COVID-19 impairs the ability of resting memory T cells to switch from using OXPHOS to using glycolysis and FAO upon antigen re-encounter, which is critical to mount effective memory responses [29]. We think that the discordance between increased glycolysis usage in memory T cells at the population level but decreased glycolysis usage in antigen specific T cells represents a compensatory mechanism by which memory T cells attempt to rectify the metabolic deficit present in antigen specific cells. In a broader sense, this may mean that prior COVID-19 infection can prevent the antigen-specific memory cell from turning on catabolic pathways (glycolysis, FAO) to generate energy which may leave individuals more vulnerable to other infections in the medium to long-term.

Patients who suffer from post-COVID immune suppression would also greatly benefit from discovery of specific treatment options. Here, we demonstrate that exposure to commonly available drugs that stimulate mitochondrial function, metformin and ubiquinol, can partially correct post-COVID deficits in memory T cell metabolism. Metformin increases T cell function in numerous ways: it can decrease T cell senescence [45], improve T cell fitness by reducing hypoxic damage [46], and reprogram T cell metabolism to promote tumor clearance [47]. The mitochondrial complex III activator ubiquinol is a potent antioxidant which limits damage from ROS to promote T cell survival [48]. Perhaps most importantly, both are inexpensive commonly available drugs which may help to rectify post-COVID suppression of T cell memory responses.

Overall, our study showcases the durability of post-COVID immunometabolic remodeling of antigen specific memory T cells in otherwise healthy convalescents. It will be critical in future studies to evaluate the extent to which SARS-CoV-2 viral persistence may contribute to memory T cell dysfunction in larger cohorts, particularly in vulnerable patient populations such as those undergoing a major intervention (e.g., surgery) requiring large catabolic reserves.

### Limitations of study

The major limitation of this study is its sample size. Of the original 31 patients we enrolled, we were only able to collect data from subsets of the group for each figure due to sample availability constraints (ex. limited numbers of PBMCs isolated, study subjects contracted COVID-19 at different times). This made it difficult to include all samples in all experiments, though we strove to increase our sample size as much as possible. In particular, our sample size comes down to our decision to include matched samples only from individuals who were initially COVID-naïve, or unexposed. As the pandemic went on, identifying and enrolling unexposed individuals became more difficult and is almost impossible currently. Indeed, for experiments assessing T cell functional capacity and cytokine production (S10 Fig), we were limited to a small cohort of patients who were infected multiple times with SARS-CoV-2 and were not necessarily naïve at baseline due to sample constraints. While supporting our post-COVID memory T cell dysfunction hypothesis, these were not ideally matched samples to the rest of the study. We think future studies can build on our findings with larger sample sizes if they are not limited to COVID-naïve patients. Another minor limitation is the use of peptide pools comprising a single antigen from IAV, SA, or VZV which may not capture the full breadth of immune responses to the respective pathogens. Finally, 2 patients with autoimmune comorbidities were on immunomodulatory medications at sample collection (though they had been on the treatments for years both before and after their first SARS-CoV-2 infection, which somewhat controls for their immunosuppressive effects). This may be an additional confounder when interpreting our results.

## Methods

### Ethics statement

Ethical approval for this study was obtained from the Northwestern University Institutional Review Board. All research on human subjects was conducted in accordance with the principles outlined in the Declaration of Helsinki and in accordance with institutional statutory requirements. All participants gave formal written consent to participate in the study and for their anonymized data to be published.

### Study participant information

We enrolled 31 healthy adult volunteers from Chicago, with an average age of 35. All participants provided informed consent and were recruited under IRB# STU00212583 from Northwestern University between 2021–2025. Subjects were enrolled after receiving COVID-19 mRNA vaccination and confirmed negative for SARS-CoV-2 by assessing antibody titers for SARS-CoV-2 nucleocapsid (Fig 1C). Participants were vaccinated with the primary series of either the Pfizer BNT162B2 or Moderna mRNA-1273 mRNA vaccines during the course of the study. Blood samples were collected every two weeks during the first month, then monthly for 6 months. This sampling schedule was repeated following booster vaccination. Subjects were also instructed to report COVID-19 infection - following a positive PCR or antigen test - at any point within a year of vaccination. This approach enabled us to collect matched blood samples before and after infection, which were used for downstream assays following PBMC isolation. Additional demographic information can be found in Fig 1D. Comorbidities were self-reported and diagnosed prior to SARS-CoV-2 infection/vaccination.

### PBMC and plasma collection

Peripheral blood (30 mL) was collected from study participants both before and after SARS-CoV-2 infection. Samples were processed using a standard Ficoll density gradient protocol. Blood was diluted 1:1 with sterile 1x PBS and carefully layered (up to 30 mL) over 15 mL of Histopaque-1077 in 50 mL conical tubes. Tubes were centrifuged at 200 g for 30 minutes at room temperature. The upper plasma layer was aspirated and discarded, and the PBMC layer was carefully isolated without disturbing the RBC layer. Cells were cryopreserved in FBS + 10% DMSO at a concentration of 10–20 million cells/mL. Samples were frozen at –80°C and transferred to liquid nitrogen for long-term storage.

### Peptide antigens used in cell stimulation

Peptide arrays for antigen stimulation were obtained from BEI Resources or from GenScript. Three arrays were used: Influenza Virus A (H1N1) Neuraminidase Protein, VZV Orf4 Protein, and *Staphylococcus aureus*-derived Hemolysin A. The Influenza virus peptide array consisted of 13–17mers with 11aa overlaps spanning the neuraminidase (NA) protein (BEI Resources). The VZV Orf4 peptide array consisted of 20mers with 10aa overlaps. Finally, the Hemolysin A peptide array consisted of peptides between 15 and 16mers with 10aa (both from GenScript). All three antigens were chosen for their ability to induce potent CD4 T-cell responses [49–51].

### Antibody ELISA

Antigen-specific antibody titers were measured by ELISA as described previously [14]. In brief, 96-well flat-bottom MaxiSorp plates (Thermo Scientific) were coated with 1 µg/mL of one of the following recombinant proteins: SARS-CoV-2 Nucleocapsid, Influenza A Hemagglutinin, or hemolysin A from Staphylococcus aureus. Plates were then incubated at 4 °C for 48 hours and washed three times with wash buffer (PBS + 0.05% Tween 20). Blocking was performed with blocking solution (PBS + 0.05% Tween 20 + 2% bovine serum albumin), for 4 hr at room temperature. 6 µl of sera was added to 144 µl of blocking solution in the first column of the plate, 1:3 serial dilutions were performed until row 12 for each sample, and plates were incubated for 60 min at room temperature. Plates were washed three times with wash buffer followed by addition of secondary antibody conjugated to horseradish peroxidase, goat anti-human IgG (H + L) (Jackson ImmunoResearch) diluted in blocking solution (1:1000) and 100 µl/well was added and incubated for 60 min at room temperature. After washing plates three times with wash buffer, 100 µl/well of Sure Blue substrate (SeraCare) was added for 1 min. Reaction was stopped using 100 µl/well of KPL TMB Stop Solution (SeraCare). Absorbance was measured at 450 nm using a Spectramax Plus 384 (Molecular Devices). SARS-CoV-2 Nucleocapsid protein produced at the Northwestern Recombinant Protein Production Core by Dr. Sergii Pshenychnyi using plasmids that were produced under HHSN272201400008C and obtained from BEI Resources, NIAID, NIH: Vector pCAGGS containing the SARS-related coronavirus 2, NR-52309, nucleocapsid gene NR-53507. Purified H1N1 Hemagglutinin protein was obtained from R&D Biosystems. Recombinant *S. aureus* hemolysin A was purchased from LS Bio. VZV glyocoprotein E ELISA was purchased from ACRO Biosystems and the assay conducted according to manufacturer’s protocol.

### IFN-γ ELISPOT

Multiscreen-IP plates (Millipore-Sigma) were coated overnight at 4°C with 2μg/mL anti-IFN-γ (clone 1-D1K, Mabtech) washed with sterile PBS, and blocked with complete RPMI-10% FBS. PBMC isolated from Neuro-PASC, COVID convalescent, and healthy control subjects were used either freshly isolated or after thawing and resting overnight in media containing 10ng/μL recombinant human IL-15 (Peprotech) at 37°C, 5% CO_2_. Cells were then plated at a concentration of 2.5x10^5^ cells/well in 200μL of media and stimulated with the indicated antigen mixtures from SARS-CoV-2 at a concentration of 2μg/mL in complete RPMI medium containing 5% human AB serum (Sigma-Aldrich) and 5ng/mL IL-15. Plates were incubated at 37°C, 5% CO_2_ for 20h and washed 5x with dH_2_O and PBS-0.05% Tween-20 (PBS-T). 2μg/mL biotinylated IFN-γ (clone 7-B6-1, Mabtech) diluted in PBS-10% FBS (PBS-F) was added to the respective wells and plates were incubated for 1.5h at RT. Plates were subsequently incubated for 40 minutes at RT in streptavidin-alkaline phosphatase in PBS-F (Jackson ImmunoResearch) was added after washing plates 5x in PBS-T. ELISPOT plates were developed using an Alkaline Phosphatase Conjugate Substrate Kit according to manufacturer’s instructions (Bio-Rad Laboratories, Carlsbad, CA). IFN-γ producing cells were quantified using an ImmunoSpot plate reader (Cellular Technologies, Ltd., Shaker Heights, OH).

### PBMC stimulation, T cell purification, and bulk RNA-Seq

Matched pre- and post-COVID PBMC samples from 16 individuals were randomly chosen for bulk RNA-Seq experiments. Cells were stimulated for 24h with 2µg/mL VZV-Orf4 peptides before cell isolation. CD4 + CD45RO+ memory T cells were isolated by negative selection using the CD4 memory T cell isolation kit from Miltenyi Biotec. Cells were lysed and RNA extracted using the RNEasy Mini Kit (Qiagen). Library preparation was performed using the Illumina Stranded Total RNA-Seq Library Prep and sequenced using the Illumina NovaSEQ X Plus (Northwestern University Genomics Core Facility). Data were analyzed using the SCALES method from the Raman Lab 18.

### Flow cytometry

For assessment of mitochondrial function, PBMCs were stimulated with IAV, SA, and VZV antigens at 2µg/mL for 16-18h. Vehicle controls were media alone. Cells were then stained with a surface antibody cocktail consisting of the following markers: CD3-BUV395, CCR7-BUV737, CD27-BUV805, CD8-V510, CD4-BV711, CD45RA-BV570, CD45RO-BV605, ICOS-AF700, CD69-BB700, PD1-BV421, CD137-BUV661, CD134-BV650 (all from BD Biosciences), CD14-APCFire 780, KLRG1-APCFire 810, Zombie UV live-dead, and CXCR5-PE-CF594 (all from Biolegend). For mitochondrial dye staining, MitoSOX Green, TMRM, and Mitotracker Red were purchased from Thermo Fisher. Cells were washed after surface staining and resuspended in warm PBS before the addition of mitochondrial dyes at the appropriate concentrations. All experiments included unstimulated cells stained with surface antibodies without addition of mitochondrial dyes. Cells were incubated for 30min at 37˚C, 5%CO_2_ in the dark before being washed in PBS and run through flow cytometry. Samples were acquired on a BD FACS Symphony 5 laser spectral flow cytometer at the Northwestern University Flow Cytometry Core Facility.

For intracellular cytokine staining, PBMCs were left unstimulated or were stimulated at a 1:1 bead to cell ratio with αCD3/CD28 human T cell activator beads (Invitrogen) for 24h. Beads were removed prior to intracellular cytokine staining by magnet. Cells were stained with a surface cocktail for 30 min. at 4˚C, fixed/permeabilized with Biolegend True Nuclear Fixation and Permeabilization buffer, and then stained with intracellular antibodies for 45min. at room temperature in the dark (antibody panel in S1 Table). Samples were acquired on a Cytek Aurora 5 laser spectral flow cytometer at the University of Chicago Antibody and Cytometry Core Facility.

For Met-Flow, our staining procedure was adapted from Ahl et al. [26]. Based on previous studies, we performed a 20-hour incubation with antigens + /- metformin 10µM [52] or ubiquinol 5 µM [53], PBMCs were washed once with FACS wash buffer. Vehicle controls were media alone. Each well was stained with Live/Dead Blue dye before sequential surface and intracellular staining as above. Antibody panel information in S2 Table. Samples were acquired on a Cytek Aurora 5 laser spectral flow cytometer at the University of Chicago Antibody and Cytometry Core Facility.

### Cell stimulation and multiplex secreted cytokine detection

Pre- and post-COVID PBMC samples were left unstimulated or stimulated with αCD3/CD28 beads as above and incubated for 72h. Cells were centrifuged and supernatants were isolated for multiplex cytokine detection using a Legendplex assay (Biolegend). Targets probed were IL-6, IFN-γ, TNFα, IL-10, IL-17A, and IL-2.

### Plasma polar metabolomic profiling using liquid chromatography/mass spectrometry (LC/MS)

30µL of plasma was mixed with 120µL of ice-cold 75/25 acetonitrile/methanol containing 0.1% formic acid, followed by continuous mixing for 5 mins at 4°C (using Thermomixer 2000 rpm) and incubate on ice for 20mins, spun down at 18,000g for 30mins at 4°C and the supernatant was transferred to an autosampler vial for LC-MS analysis. The metabolite separation was performed using the Thermo Scientific Vanquish Horizon UHPLC system and iHILIC-(P) Classic (2.1x150 mm, 5 µm; part # 160.152.0520; HILICON AB) column. The high-resolution Orbitrap IQ-X Tribrid mass spectrometer (Thermo Scientific) with a H-ESI probe operating in switch polarity was used to detect and quantify the metabolite levels. The mobile phase A(MPA) was 20 mM ammonium bicarbonate at pH 9.6, adjusted by ammonium hydroxide addition and MPB was acetonitrile. The column temperature, injection volume, and the flow rate were 40°C, 2 µL, and 0.2mL/minute, respectively. The chromatographic gradient was 0 minutes: 85% B, 0.5 minutes: 85% B, 18 minutes: 20% B, 20 minutes: 20% B, 20.5 minutes: 85% B and 28 minutes: 85% B. MS parameters were as follows: Acquisition range of 70–1000 m/z at 60K resolution, spray voltage:3600V for positive ionization and 2800 for negative ionization modes, sheath gas: 35, auxiliary gas: 5, sweep gas: 1, ion transfer tube temperature: 250°C, vaporizer temperature: 350°C, AGC target: 100%, and a maximum injection time of 118 ms. Poole QC samples were injected intermittently to determine the reproducibility of the detection and metabolite stability over time. Data acquisition was done using the Xcalibur software (Thermo Scientific) and data analysis was performed using Compound Discoverer 3.3 (± 5 ppm) & Skyline software [54]. Metabolite identification was done by matching the retention time and MS/MS fragmentation to the in-house database generated using the commercially available reference standards. In the data table, the “RT+MS/MS” indicates the matching retention time & MS/MS, “RT”-indicates the only matching retention time and doesn’t have MS/MS while the MS/MS is for carnitine species identified based on the 85.0281 fragment.

### Quantitative PCR to detect SARS-CoV-2 in PBMCs

RNA from PBMCs was isolated after antigen stimulation using the QIAamp DSP Viral RNA Mini Kit (Qiagen; Cat no./ ID. 61904) and RNA concentrations and quality were measured using the NanoDrop 2000 (ThermoFisher Scientific). Specific components from the NEB Luna Probe one-step RT-qPCR kit (New England Biolabs; cat. # M3029S, NEB Luna Probe One-Step RT-qPCR 4X Mix with UDG), SARS-CoV-2 Neo Assay Kit (Qiagen; Cat no./ ID. 222115, SARS-CoV-2 Neo Assay 20x primer/probe mix), and QIAprep&amp Viral RNA UM Kit (cat no. 221413, 221415, and 221417, human sampling IC assay; Mat. No. 1125243 and Lot No. 178039830) were utilized to prepare the assay mix of all components except for < 1 μg (total RNA) of the RNA template. To mix samples, gentle pipetting and inverting were completed. 18 µL of assay mix was pipetted first into each well. Following, samples had less than 1 µg of total RNA added to each well for up to 2 µL of volume, 2 µL total of positive controls (Orf1a/Orf1b - ROX and N1/N2 – FAM) or nuclease-free water as the negative control for a total of 20 µL volume reaction. Samples were run in technical duplicates using the QuantStudio 3 Flex Real-Time PCR Systems (Thermo Fisher Scientific) in a 96-well plate with a total reaction volume of 20 µL. Data analysis was conducted using QuantStudio Design & Analysis 2.x Software and visualized in a table using Ct/Cq values.

### Boltzmann brain analysis

The matrix input into the Boltzmann Brain constituted X rows defining sample and Y columns defining a cellular feature, where each feature was either the percentage of cells in the population expressing a specified protein marker (Fig 4J-4K), or the geometric mean fluorescence intensity (gMFI) of protein expression on a per-cell basis derived from flow cytometry data analysis (Fig 5D-5E). In each case, the rows of the matrix were labeled by a metadata ‘Group’ variable constituting either Pre-COVID or Post-COVID. The Boltzmann Brain first performs eigendecomposition of the matrix, creating a spectrum of principal components. The Brain then computes the mutual information (MI) between all pairs of principal components. This calculation is performed in the following way. Each principal component is characterized by a set of projections. If two principal components share a non-random pattern of projections, their MI is statistically significant.

Computing the MI for all pairs of principal components creates a matrix where each row and column is a single principal component, and each entry is the MI between two principal components. Hierarchical clustering of this matrix defines a layered graph where at each layer, two principal components are connected if they share MI with each other. By definition, the bottom of this graph is each principal component unconnected to any other principal component. Also by definition, the top of the graph connects all principal components together. Simultaneously, the Boltzmann Brain also computes the MI of all principal components with the metadata variable of Group. As an intuitive rationale for this computation, if a specific principal component splits pre-COVID from post-COVID samples, then this principal component shares a high-degree of MI with the meta-data variable of ‘Group’. The computation for MI as well as all code related to computing MI between principal components and meta-data variables has been described in previous publications (see Zaydman *et al*., eLife 2022, PMID 35976223; and Doran *et al*., Cell Systems 2025, PMID 39826551). (The code for performing Singular Value Decomposition and hierarchical clustering is manifest by standard R packages.)

Briefly, MI is the difference between the intrinsic uncertainty in a measurement bounded by *a* to *b*, $H\left(\rho^{a:b}\right)$, and the uncertainty computed given a benchmark *c* (e.g., ‘Group’ variable in this case), $H\left(\rho^{a:b}|c\right)$.


$$\begin{array}{c} I\left(\rho^{a:b},c\right)=\;H\left(\rho^{a:b}\right)-H\left(\rho^{a:b}|c\right) \end{array}$$

The uncertainty principal component projections given knowledge of a benchmark is evaluated in the following way. A benchmark *c* can be either ‘1’ if two variables share the benchmark (i.e., two samples are both pre-COVID samples or post-COVID samples) or ‘0’ if two variables do not share the benchmark. The ‘conditional entropy’ is the differential entropy conditioned on knowledge of the probability distribution of benchmarks. So, $H\left(\rho^{a:b}|c\right)$ is mathematically defined as


$$\begin{array}{c} H\left(\rho^{a:b}|c\right)=p(c=\text{1})H\left(\rho^{a:b}|c=\text{1}\right)+p(c=\text{0})H\left(\rho^{a:b}|c=\text{0}\right) \end{array}$$

where *p*(c = 1) and *p*(c = 0) are the probability of observing a ‘1’ or ‘0’ in *c* respectively; and $H\left(\rho^{a:b}|c=\text{1}\right)$ and $H\left(\rho^{a:b}|c=\text{0}\right)$ are the differential entropies of spectral correlations conditioned upon variables that share (*c* = 1) and do not share (*c* = 0) a benchmark respectively.

Network graphs in Fig 4L and Fig 5D display the network graphs of mutual information between all pairs principal components at the first layer where any principal component in the networks shares MI with the meta-data variable of ‘Group’. Each numbered node is a principal component, any numbered node that is colored is within a network of connected principal components sharing MI with each other; any numbered node that is white indicates principal components that do not share MI with each other. Additionally, in the network graphs of Fig 4L and Fig 5D, meta-data variables are shown in Red with arrows pointing to the ‘Group’ meta-data variable.

Once the network graph is created, a UMAP embedding is constructed across all principal components in the MI graph. This UMAP represents a high-dimensional embedding where principal components of the data are now related to the meta-data variable of ‘Group’. Features are then extracted that are associated with different ‘Group’ categories within this embedding. These are the Z-score plots shown in Fig 4M and Fig 5E. As an example, in Fig 5E, MFI(CPT1A) separates Pre-COVID from Post-COVID samples considering gMFI as the feature space over which the embedding was learned.

### Statistical analysis

All data were analyzed in either GraphPad Prism 10 or using R/Python. All flow cytometry data were analyzed using FlowJo v10.

### Schematic figures

Experimental schematics (Figs 1C, 1E, 2A, 6, S8) were made in Biorender and published under CC-BY licensing via Biorender premium subscription.

## Supporting information

S1 Fig T cell responses to SARS-CoV-2 vaccination are quantitatively similar in those vaccinated before any SARS-CoV-2 infection and those previously infected prior to vaccination. A.) PBMCs from naïve patients included in this study (healthy controls, HC) were assayed for IFN-γ responses to SARS-CoV-2 vaccination. T cell responses were quantitatively compared to vaccine-elicited IFN-γ production in those vaccinated after infection (COVID convalescents, CC). Spike-specific IFN-γ production was assessed pre-vaccination (visit 0) and approximately every month thereafter until 6 months post-vaccination (visit 4). Spike-specific IFN-γ responses were significantly elevated in CC relative to HC at baseline, but stabilized thereafter. This suggests that infection + vaccination similarly increase clonal expansion of Spike-specific T cells and therefore, SARS-CoV-2 infection would not overwhelm T cell responses to other pathogens simply due to virus-specific T cell clonal expansion. B.) IFN-γ ELISPOT showing limited expansion of T cell response to SARS-CoV-2 Nucleocapsid protein post-infection (n = 2 matched samples included in this study). *p < 0.05 by two-tailed Student’s t Test.
(DOCX)

S2 Fig Pre-COVID antibody titers, VZV/SA/IAV antibody titers, antigen descriptions. A.) All subjects were negative for exposure to COVID-19 by anti-SARS-CoV-2 N protein (Nucleocapsid) serology. -ctrl: pre-2019 samples. + ctrl: patients diagnosed with long COVID after positive PCR test. B.) 93% of study subjects were positive for VZV antibody titers upon enrollment. All were confirmed to have been vaccinated or exposed to natural infection in childhood. -ctrl and +ctrl were provided with the manufacturer’s kit (ACRO Biosystems). C.) Description of antigens used in stimulation from VZV, IAV, and SA. D.) All subjects were positive for IAV and SA IgG by serology. Horizontal lines in B & D represent limit of detection.
(DOCX)

S3 Fig CD4 memory T cell purity after magnetic bead negative selection. PBMCs were stimulated with VZV-Orf4 peptides and CD4 + CD45RO+ cells were purified by negative selection using a Miltenyi CD4 memory T cell purification kit. Samples were approximately 92% CD3 + T cells, of which 80% were CD4 + .
(DOCX)

S4 Fig PCA analysis of pre- and post-COVID RNA-Seq data uncover batch and age effects as main global covariates. A.) Traditional PCA analysis identified batch effects as principal components describing statistical variation. B.) Age effects are similarly identified among the top PCs and are partially co-linear with batch effects. The confounders present in these global covariates were resolved using the SCALES method described in the results.
(DOCX)

S5 Fig Flow cytometry gating strategies. A.) Strategy for gating CD4 and CD8 memory T cells. B.) Fluorescence minus one (FMO) gating for Met-Flow enzymes.
(DOCX)

S6 Fig Gating strategy for AIM+ CD4 and CD8 memory T cells. A.) Boolean gating strategy was applied for identifying AIM + CD4 + CD45RO + T cells. Cells were gated on CD69/CD137/CD154/CD134+ as in the top row before applying Boolean and/or gating (combination gates) in Flowjo. The 6 AIM combinations shown were concatenated together and metabolic enzyme expression analyzed within the concatenated population. B.) Gating strategy for AIM + CD8 memory T cells.
(DOCX)

S7 Fig Active mitochondrial mass is similar in antigen-stimulated pre- and post-COVID AIM+ memory T cells. A.) Mitochondrial mass (MTT) is similar in pre- and post-COVID AIM + CD4 + CD45RO+ memory T cells, with the exception of SA stimulated T cells. B.) MTT is similar across all antigen stimulation conditions in AIM + CD8 + CD45RO+ memory T cells.
(DOCX)

S8 Fig Diagram of intracellular metabolic pathways interrogated in Met-Flow. Metabolic enzyme targets probed in flow cytometry are in red boxes. Created in BioRender. Visvabharathy, L. (2026) https://urldefense.com/v3/__https://BioRender.com/76sbzcs__;!!MyIu0v6UfBA57LoN!7vUZ9kyk3qCkLXcU3eXdJyQxGv2nwFA5QQZJ-goxoexxrJnzzhDcR6ClzctJR87xgJcEsTzqLlxq35qRbJ_DiMHMYqjUYPM29xQ$.
(DOCX)

S9 Fig Met-Flow enzyme expression levels are similar in unstimulated pre- and post-COVID samples in memory T cells. A.) %CD4 + CD45RO + T cells positive for specified marker. B.) %CD8 + CD45RO + T cells positive for specified marker. ****p < 0.001 by paired Student’s t test.
(DOCX)

S10 Fig Increased FoxP3+ Tregs in post-COVID samples at baseline, and diminished capacity for T cell production of IFN-γ after αCD3/CD28 activation. A.) Demographic table for samples used in this figure. We obtained matched samples before and after an individual’s first infection as in the rest of the paper for n = 2 patients. For the remaining 7, we obtained PBMC samples before and after their 2^nd^ or greater infection. B.) FoxP3 + CD4 + T_regs_ are elevated in post-COVID samples at baseline. C.) Increased baseline production of IFN-γ and IL-6 in unstimulated post-COVID PBMCs. D.) Significant decrease in T cell production of IFN-γ after αCD3/CD28 stimulation of the T cell receptor (TCR). IFN-γ was detected in culture supernatants after 72h incubation. E.) Elevated PD1 + TCF1 + precursors of exhausted CD8 memory T cells (T_pex_) in post-COVID samples. F.) αCD3/CD28 stimulation induces expression of the transcription factor TCF1 in pre-COVID CD4 memory T cells, indicating higher proliferative capacity relative to activated post-COVID T cells. *p < 0.05 by two-tailed paired Wilcoxon test. B, E, F: 24h incubation of PBMCs. C, D: 72h incubation of PBMCs; supernatants were assayed for cytokine production. B, F: n = 7; C: n = 10; D, E: n = 8.
(DOCX)

S11 Fig Exposure to metformin or ubiquinol does not alter cell viability in culture. PBMCs from matched pre- and post-COVID samples were stimulated with VZV peptides in the presence or absence of Metformin 10µM or Ubiquinol 5µM for 18-20h. Cell viability was measured by FACS staining with Zombie Blue (Biolegend). Viable cells are Zombie Blue negative. No difference in cell viability was found between treatment groups. N = 16 samples.
(DOCX)

S12 Fig RT-qPCR cannot detect residual SARS-CoV-2 in post-COVID PBMCs. RT-qPCR for SARS-CoV-2 Nucleocapsid RNA within RNA isolated from post-COVID PBMCs. LoD: limit of detection.
(DOCX)

S1 Table Antibody panel for intracellular cytokine staining. (DOCX)

S2 Table Antibody panel for MetFlow experiments. (DOCX)

S1 Data Underlying data for main Figs 1–4. (DOCX)
