# Supplementary material for: Post-COVID impairment of T cell responses to community-acquired pathogens can be modified by activating cellular metabolism
Source: PLoS Pathog. 2026 Jul 30;22(7):e1013860. doi: 10.1371/journal.ppat.1013860 (PMC13441075; doi:10.1371/journal.ppat.1013860)
Supplement: Supplementary file 1 [file ppat.1013860.s017.pdf]

Reviewer comments:

The study by Alves Esposito et al. Analyzed donor-matched blood samples from 31 COVID-naïve individuals who later contracted COVID-19, to track how influenza A (IAV), Staphylococcus aureus (SA), and Varicella-zoster virus (VZV)-stimulated T cell responses were affected by COVID infection. They demonstrated a decrease in mitochondrial function in antigen-specific T cells post-COVID. They also found decrease in gene expression linked to T cell activation but increased mitochondrial redox pathway usage in post-COVID CD4 memory T cells. However, mitochondrial flux and reactive oxygen species production were limited in many post-COVID-19 antigen-specific T cells after stimulation with IAV, SA, and VZV. Post-COVID plasma was depleted in carnitine and TCA cycle species essential for activating fatty acid oxidation, correlating with disrupted T cell metabolism and mobilization of glycolysis, fatty acid metabolism, and oxidative phosphorylation pathways. Metabolic activation with metformin and ubiquinol partially restored T cell catabolism after COVID. Overall findings from their study suggest that SARS-CoV2 infection may have lasting effects on T cell metabolism upon encountering common pathogens.

Overall, the manuscript has improved substantially compared to the previous version. However here are some points that need to be considered.

Minor:

- The abstract is poorly written and requires improvement for better clarity and impact.
- What exclusion criteria were applied when selecting donors beyond being naïve or then having SARS-CoV-2 infection? Were any immunocompromised individuals included in the study? Clarifying this information is important for interpreting the data. Please consider adding a paragraph in the Discussion to address this point (or in limitation section).
- The discussion should present the findings concisely. I recommend that the authors revise the discussion section accordingly.

The authors have fully addressed all the comments and suggestions. Overall, this study will be of great interest to readers.
