# Supplementary material for: Post-COVID impairment of T cell responses to community-acquired pathogens can be modified by activating cellular metabolism
Source: PLoS Pathog. 2026 Jul 30;22(7):e1013860. doi: 10.1371/journal.ppat.1013860 (PMC13441075; doi:10.1371/journal.ppat.1013860)
Supplement: Supplementary file 2 [file ppat.1013860.s001.docx]

**Figure S1: T cell responses to SARS-CoV-2 vaccination are quantitatively similar in those vaccinated before any SARS-CoV-2 infection and those previously infected prior to vaccination.**

A.) PBMCs from naïve patients included in this study (healthy controls, HC) were assayed for IFN-γ responses to SARS-CoV-2 vaccination. T cell responses were quantitatively compared to vaccine-elicited IFN-γ production in those vaccinated after infection (COVID convalescents, CC). Spike-specific IFN-γ production was assessed pre-vaccination (visit 0) and approximately every month thereafter until 6 months post-vaccination (visit 4). Spike-specific IFN-γ responses were significantly elevated in CC relative to HC at baseline, but stabilized thereafter. This suggests that infection + vaccination similarly increase clonal expansion of Spike-specific T cells and therefore, SARS-CoV-2 infection would not overwhelm T cell responses to other pathogens simply due to virus-specific T cell clonal expansion. B.) IFN-γ ELISPOT showing limited expansion of T cell response to SARS-CoV-2 Nucleocapsid protein post-infection (n=2 matched samples included in this study). *p<0.05 by two-tailed Student’s t Test.
