# Supplementary material for: Post-COVID impairment of T cell responses to community-acquired pathogens can be modified by activating cellular metabolism
Source: PLoS Pathog. 2026 Jul 30;22(7):e1013860. doi: 10.1371/journal.ppat.1013860 (PMC13441075; doi:10.1371/journal.ppat.1013860)
Supplement: Supplementary file 3 [file ppat.1013860.s002.docx]

**Figure S2: Pre-COVID antibody titers, VZV/SA/IAV antibody titers, antigen descriptions.**

A.) All subjects were negative for exposure to COVID-19 by anti-SARS-CoV-2 N protein (Nucleocapsid) serology. -ctrl: pre-2019 samples. +ctrl: patients diagnosed with long COVID after positive PCR test. B.) 93% of study subjects were positive for VZV antibody titers upon enrollment. All were confirmed to have been vaccinated or exposed to natural infection in childhood. -ctrl and +ctrl were provided with the manufacturer’s kit (ACRO Biosystems). C.) Description of antigens used in stimulation from VZV, IAV, and SA. D.) All subjects were positive for IAV and SA IgG by serology. Horizontal lines in B & D represent limit of detection.
