# Supplementary material for: Post-COVID impairment of T cell responses to community-acquired pathogens can be modified by activating cellular metabolism
Source: PLoS Pathog. 2026 Jul 30;22(7):e1013860. doi: 10.1371/journal.ppat.1013860 (PMC13441075; doi:10.1371/journal.ppat.1013860)
Supplement: Supplementary file 4 [file ppat.1013860.s003.docx]

**Figure S3: CD4 memory T cell purity after magnetic bead negative selection.**

PBMCs were stimulated with VZV-Orf4 peptides and CD4+CD45RO+ cells were purified by negative selection using a Miltenyi CD4 memory T cell purification kit. Samples were approximately 92% CD3+ T cells, of which 80% were CD4+.
