# Supplementary material for: Post-COVID impairment of T cell responses to community-acquired pathogens can be modified by activating cellular metabolism
Source: PLoS Pathog. 2026 Jul 30;22(7):e1013860. doi: 10.1371/journal.ppat.1013860 (PMC13441075; doi:10.1371/journal.ppat.1013860)
Supplement: Supplementary file 5 [file ppat.1013860.s004.docx]

**Figure S4: PCA analysis of pre- and post-COVID RNA-Seq data uncover batch and age effects as main global covariates.**

A.) Traditional PCA analysis identified batch effects as principal components describing statistical variation. B.) Age effects are similarly identified among the top PCs and are partially co-linear with batch effects. The confounders present in these global covariates were resolved using the SCALES method described in the results.
