# Supplementary material for: Post-COVID impairment of T cell responses to community-acquired pathogens can be modified by activating cellular metabolism
Source: PLoS Pathog. 2026 Jul 30;22(7):e1013860. doi: 10.1371/journal.ppat.1013860 (PMC13441075; doi:10.1371/journal.ppat.1013860)
Supplement: Supplementary file 6 [file ppat.1013860.s005.docx]

**Figure S5: Flow cytometry gating strategies.**

A.) Strategy for gating CD4 and CD8 memory T cells. B.) Fluorescence minus one (FMO) gating for Met-Flow enzymes.
