# Supplementary material for: Post-COVID impairment of T cell responses to community-acquired pathogens can be modified by activating cellular metabolism
Source: PLoS Pathog. 2026 Jul 30;22(7):e1013860. doi: 10.1371/journal.ppat.1013860 (PMC13441075; doi:10.1371/journal.ppat.1013860)
Supplement: Supplementary file 7 [file ppat.1013860.s006.docx]

**Figure S6: Gating strategy for AIM+ CD4 and CD8 memory T cells.**

A.) Boolean gating strategy was applied for identifying AIM+ CD4+CD45RO+ T cells. Cells were gated on CD69/CD137/CD154/CD134+ as in the top row before applying Boolean and/or gating (combination gates) in Flowjo. The 6 AIM combinations shown were concatenated together and metabolic enzyme expression analyzed within the concatenated population. B.) Gating strategy for AIM+CD8 memory T cells.
