# Supplementary material for: Post-COVID impairment of T cell responses to community-acquired pathogens can be modified by activating cellular metabolism
Source: PLoS Pathog. 2026 Jul 30;22(7):e1013860. doi: 10.1371/journal.ppat.1013860 (PMC13441075; doi:10.1371/journal.ppat.1013860)
Supplement: Supplementary file 8 [file ppat.1013860.s007.docx]

**Figure S7: Active mitochondrial mass is similar in antigen-stimulated pre- and post-COVID AIM+ memory T cells.**

A.) Mitochondrial mass (MTT) is similar in pre- and post-COVID AIM+ CD4+CD45RO+ memory T cells, with the exception of SA stimulated T cells. B.) MTT is similar across all antigen stimulation conditions in AIM+CD8+CD45RO+ memory T cells.
