# Supplementary material for: Post-COVID impairment of T cell responses to community-acquired pathogens can be modified by activating cellular metabolism
Source: PLoS Pathog. 2026 Jul 30;22(7):e1013860. doi: 10.1371/journal.ppat.1013860 (PMC13441075; doi:10.1371/journal.ppat.1013860)
Supplement: Supplementary file 9 [file ppat.1013860.s008.docx]

**Figure S8: Diagram of intracellular metabolic pathways interrogated in Met-Flow.**

Metabolic enzyme targets probed in flow cytometry are in red boxes.
