# Supplementary material for: Post-COVID impairment of T cell responses to community-acquired pathogens can be modified by activating cellular metabolism
Source: PLoS Pathog. 2026 Jul 30;22(7):e1013860. doi: 10.1371/journal.ppat.1013860 (PMC13441075; doi:10.1371/journal.ppat.1013860)
Supplement: Supplementary file 10 [file ppat.1013860.s009.docx]

**Figure S9: Met-Flow enzyme expression levels are similar in unstimulated pre- and post-COVID samples in memory T cells.**

A.) %CD4+CD45RO+ T cells positive for specified marker. B.) %CD8+CD45RO+ T cells positive for specified marker. ****p<0.001 by paired Student’s t test.
