# Supplementary material for: Post-COVID impairment of T cell responses to community-acquired pathogens can be modified by activating cellular metabolism
Source: PLoS Pathog. 2026 Jul 30;22(7):e1013860. doi: 10.1371/journal.ppat.1013860 (PMC13441075; doi:10.1371/journal.ppat.1013860)
Supplement: Supplementary file 11 [file ppat.1013860.s010.docx]

**Figure S10: Increased FoxP3+ Tregs in post-COVID samples at baseline, and diminished capacity for T cell production of IFN-γ after αCD3/CD28 activation.**

A.) Demographic table for samples used in this figure. We obtained matched samples before and after an individual’s first infection as in the rest of the paper for n=2 patients. For the remaining 7, we obtained PBMC samples before and after their 2^nd^ or greater infection. B.) FoxP3+ CD4+ T_regs_ are elevated in post-COVID samples at baseline. C.) Increased baseline production of IFN-γ and IL-6 in unstimulated post-COVID PBMCs. D.) Significant decrease in T cell production of IFN-γ after αCD3/CD28 stimulation of the T cell receptor (TCR). IFN-γ was detected in culture supernatants after 72h incubation. E.) Elevated PD1+TCF1+ precursors of exhausted CD8 memory T cells (T_pex_) in post-COVID samples. F.) αCD3/CD28 stimulation induces expression of the transcription factor TCF1 in pre-COVID CD4 memory T cells, indicating higher proliferative capacity relative to activated post-COVID T cells. *p<0.05 by two-tailed paired Wilcoxon test. B, E, F: 24h incubation of PBMCs. C, D: 72h incubation of PBMCs; supernatants were assayed for cytokine production. B, F: n=7; C: n=10; D, E: n=8.
