# Supplementary material for: Post-COVID impairment of T cell responses to community-acquired pathogens can be modified by activating cellular metabolism
Source: PLoS Pathog. 2026 Jul 30;22(7):e1013860. doi: 10.1371/journal.ppat.1013860 (PMC13441075; doi:10.1371/journal.ppat.1013860)
Supplement: Supplementary file 12 [file ppat.1013860.s011.docx]

**Figure S11: Exposure to metformin or ubiquinol does not alter cell viability in culture.**

PBMCs from matched pre- and post-COVID samples were stimulated with VZV peptides in the presence or absence of Metformin 10µM or Ubiquinol 5µM for 18-20h. Cell viability was measured by FACS staining with Zombie Blue (Biolegend). Viable cells are Zombie Blue negative. No difference in cell viability was found between treatment groups. N=16 samples.
