# Supplementary material for: Post-COVID impairment of T cell responses to community-acquired pathogens can be modified by activating cellular metabolism
Source: PLoS Pathog. 2026 Jul 30;22(7):e1013860. doi: 10.1371/journal.ppat.1013860 (PMC13441075; doi:10.1371/journal.ppat.1013860)
Supplement: Supplementary file 13 [file ppat.1013860.s012.docx]

**Figure S12: RT-qPCR cannot detect residual SARS-CoV-2 in post-COVID PBMCs.**

RT-qPCR for SARS-CoV-2 Nucleocapsid RNA within RNA isolated from post-COVID PBMCs. LoD: limit of detection.
