# Supplementary material for: Post-COVID impairment of T cell responses to community-acquired pathogens can be modified by activating cellular metabolism
Source: PLoS Pathog. 2026 Jul 30;22(7):e1013860. doi: 10.1371/journal.ppat.1013860 (PMC13441075; doi:10.1371/journal.ppat.1013860)
Supplement: Supplementary file 15 [file ppat.1013860.s014.docx]

**S2 Table. Met-Flow**

| **Antibody** | **Fluorophore** | **Dilution** | **Clone** | **Company** |
| --- | --- | --- | --- | --- |
| Zombie Blue | B550 | 1:800 | N/A | Biolegend |
| CD3 | BUV395 | 1:200 | SK7 | Biolegend |
| CD4 | BV711 | 1:200 | SK3 | Biolegend |
| CD8 | BV510 | 1:200 | SK1 | Biolegend |
| CD69 | BB700 | 1:200 | FN50 | BD |
| CD56 | BV650 | 1:200 | 5.1H11 | Biolegend |
| PD1 | BV421 | 1:200 | EH12.2H7 | Biolegend |
| HLA-DR | BV480 | 1:200 | G46-6 | BD |
| CD45RA | BV570 | 1:200 | HI100 | Biolegend |
| CD45RO | BV605 | 1:200 | UCHL1 | Biolegend |
| CD14 | PE-Fire 640 | 1:200 | 63D3 | Biolegend |
| CD16 | BV786 | 1:200 | 3G8 | Biolegend |
| CD154 | PerCP-Fire 750 | 1:200 | 24-31 | Biolegend |
| CD134 | APC-Fire 750 | 1:200 | Ber-ACT35 | Biolegend |
| CD137 | BV750 | 1:200 | 4B4-1 | Biolegend |
| CCR7 | BUV737 | 1:200 | 3D12 | BD |
| CD27 | BUV805 | 1:400 | O323 | BD |
| CPT1A* | AF350  (secondary) | 1:100 primary | 5D8G9 | ThermoFisher |
| IDH2* | AF680 | 1:100 | G-11 | Santa Cruz |
| PRDX2* | BUV615 | 1:50 | EPR5154 | BD |
| HK1* | BUV661 | 1:50 | EPR10134(B) | BD |
| TKT* | V450 | 1:100 | 7H1AA1 | BD |
| GLUT1* | AF790 | 1:100 | A-4 | Santa Cruz |
| ACAC* | AF647 | 1:200 | EPR4971 | BD |
| G6PD* | PE | 1:100 | G-12 | Santa Cruz |
| pMTOR | PE-Cy7 | 1:100 | MRRBY | ThermoFisher |
| ASS1* | Coralite594 | 1:200 | 1D1A11 | ThermoFisher |
| ATP5a* | PE-Alexa 647  (secondary) | 1:200 primary | 23GB4850 | ThermoFisher |

*****intracellular markers
