# Supplementary material for: Post-COVID impairment of T cell responses to community-acquired pathogens can be modified by activating cellular metabolism
Source: PLoS Pathog. 2026 Jul 30;22(7):e1013860. doi: 10.1371/journal.ppat.1013860 (PMC13441075; doi:10.1371/journal.ppat.1013860)
Supplement: Supplementary file 16 [file ppat.1013860.s015.docx]

**Antibody cocktails**

**Table S1. Intracellular staining antibody panel**

| **Antibody** | **Fluorophore** | **Dilution** | **Clone** | **Company** |
| --- | --- | --- | --- | --- |
| Zombie Live-dead | UV | 1:800 | N/A | Biolegend |
| CD3 | BUV395 | 1:200 | SK7 | BD |
| CD4 | BV711 | 1:200 | SK3 | Biolegend |
| CD8 | BV510 | 1:200 | SK1 | Biolegend |
| CD69 | BB700 | 1:200 | FN50 | BD |
| CD14 | PE-Dazzle 594 | 1:200 | HCD14 | Biolegend |
| Tim3 | APC-Fire750 | 1:200 | F38-2E2 | Biolegend |
| Lag3 | RY655 | 1:200 | T47-530 | BD |
| PD1 | BV421 | 1:200 | EH12.2H7 | Biolegend |
| HLA-DR | BV480 | 1:200 | G46-6 | BD |
| CD45RA | BV570 | 1:200 | HI100 | Biolegend |
| CD45RO | BV605 | 1:200 | UCHL1 | Biolegend |
| CD16 | BV786 | 1:200 | 3G8 | Biolegend |
| CD19 | AF700 | 1:200 | HIB19 | Biolegend |
| HK1* | BUV661 | 1:100 | EPR10134(B) | BD |
| IFNg* | BV650 | 1:100 | 4S.B3 | Biolegend |
| TCF1* | RY703 | 1:100 | S33-966 | BD |
| GZB* | PE-Cy7 | 1:400 | QA16A02 | Biolegend |
| FoxP3* | FITC | 1:100 | 206D | Biolegend |
| IL-6* | PE | 1:100 | MQ2-13A5 | Biolegend |
| IL10* | AF647 | 1:100 | JES3-9D7 | Biolegend |
| IL17* | BV750 | 1:100 | N49-653 | BD |
| TNFa* | APC-Fire810 | 1:200 | Mab11 | Biolegend |

*****intracellular markers
