# Supplementary material for: Post-COVID impairment of T cell responses to community-acquired pathogens can be modified by activating cellular metabolism
Source: PLoS Pathog. 2026 Jul 30;22(7):e1013860. doi: 10.1371/journal.ppat.1013860 (PMC13441075; doi:10.1371/journal.ppat.1013860)
Supplement: Supplementary file 17 [file ppat.1013860.s018.docx]

**Response to Reviewers’ comments**

We thank the Reviewers for their constructive and insightful comments and experiment suggestions which have helped us to improve our manuscript. We have addressed all their comments, and we have also revised the text of the manuscript for clarity of reading. The Reviewers’ comments appear **bolded** and are followed by our responses.

***Editorial Issues***

1. **Reconcile JCA as founder of COVIRA with no competing interests**

- We have removed the reference to JCA as founder of COVIRA because the company had nothing to do with the conceptualization, funding, or execution of the work.

2. **Boltzmann brain- The authors will provide: either (i) sufficient methodological and output disclosure to reproduce the key findings, or (ii) confirmation of key results via open, standard analytic approaches.**

- We have added a detailed explanation of Boltzmann Brain in the methods section, including references for underlying code.

3. **Soften the statements about post-COVID increases in infection rates being causally linked in CDC Wondr data**

- We have addressed this in lines 138-140.

***Major Issues-Reviewers***

**Reviewer 1:**

**1. The RNA-Seq signal in Figure 2 may be dominated by noise because the authors did not sort for Ag-specific CD4 memory T cells. Post-COVID samples may be dominated by clonal expansion of SARS-CoV-2-specific T cells.**

- This is an important point. While performing scRNA-Seq on sorted VZV-specific T cells was beyond the scope, we are able to indirectly address whether post-COVID samples were dominated by clonal expansion of SARS-specific T cells. We have added supplementary figure S1 showing vaccine-induced IFN-γ responses to SARS-CoV-2 Spike protein in naïve individuals compared to previously infected individuals. PBMC samples were obtained prior to and at various times post-vaccination. We show that while individuals infected prior to getting their first dose of mRNA vaccine began with higher IFN-γ T cell responses to Spike, the magnitude of the response equilibrated between naïve individuals and previously infected individuals by 6mo. post-vaccination. These data suggest similar clonal expansion of Spike-specific T cells in vaccinated naïve vs. vaccinated COVID-infected people, and therefore imply that our study subjects would not have increased clonal expansion of Spike-specific T cells beyond what is induced by the vaccine. We also have some data to show Nucleocapsid-specific T cell responses remain low after infection (Fig. S1B).

**2. Tone down the claims that bulk RNA-Seq differences after VZV peptide stimulation result from Ag-specific responses.**

-We have now clarified this in the text (lines 224-227).

**3. For Figure 3, it is difficult to accurately calculate MFI with low cell numbers as in AIM+ cells.**

-We had an average of 104 CD4+CD45RO+ AIM+ cells and 160 CD8+CD45RO+ AIM+ cells per sample. We think this is sufficient to accurately calculate gMFI, particularly as the data were corroborated using gMFI values in CD69+ memory T cell population containing thousands of cells per sample (data not shown).

**4. Authors observed decreased metabolic markers in CD45RO+ bulk population after covid, but this could reflect bystander effects rather than direct TCR engagement. Authors should distinguish between 2 possibilities.**

-This is true, but we limit our analysis in Figure 5 to AIM+ antigen specific T cells, which addresses this possibility. Our Figure 5 data show decreased glycolysis and FAO in Ag-specific T cells.

**5. Rescue effects in Fig. 5-Cell viability or ROS production may change after treatment with Met/Ubq. Observed metabolic enzyme increase may reflect lower activation threshold globally rather than effecting Ag-specific cells.**

-We have now added Figure S11 showing that incubation with Metformin 10µM or Ubiquinol 5µM does not alter cell viability relative to unstimulated conditions.

**6. Metabolic immobilization needs to be better contextualized w/literature on T cell exhaustion and immunosenescence. Unique to COVID-19 or caused by other infections?**

-This is now addressed in the discussion (lines 413-416).

**Reviewer 2:**

**1. The authors central claim is about impaired recall, but the data is focused on metabolic surrogates. There needs to be a direct readout of impaired function (e.g. proliferation, cytokine expression).**

-This is a very important point and we thank the reviewer for bringing it to our attention. We have now added Figure S10 showing functional impairment of IFN-γ production after TCR-dependent stimulation of post-COVID T cells as well as an expansion of CD8+ memory T cells with exhaustion markers. While we think the data compellingly support our hypothesis, we included it as a supplemental figure because we did not have enough of our original matched naïve/post first COVID infection samples remaining to assay. Instead, we had a cohort of matched patient PBMCs after their first vs. second or greater COVID infection which we used to study this question. Given that they were not part of the initial cohort on which we based the main figures, we decided to include the data as supplemental.

**2. Need to list covariates of patient COVID infection.**

-We have now added these data to the demographic table and schematic in Figure 1, including COVID clinical severity, the variant era at which patients were infected (Fig. 1E), and COVID vaccine information.

**3. Explain how Boltzmann Brain was used in greater detail to allow for data reproducibility.**

-We have now added detailed methods explaining the Boltzmann Brain algorithm, including citations where code can be found (lines 706-762).

**4. Soften claims about CDC WONDR data implying causality between the COVID pandemic and increased infection rates.**

-We have addressed this in lines 138-140.

**5. Discuss underlying mechanism behind how COVID uniquely dysregulates mitochondrial function rel. VZV/IAV.**

-We believe viral persistence is responsible, though we did not find direct evidence of it in post-COVID PBMCs, Fig. S12. Other groups have published convincing evidence of viral persistence in extra-respiratory, non-immune tissues, however, and this could impact the post-COVID T cell memory response to other pathogens. This is discussed in lines 472-475.

**6. Lines 294-296: Met/Ubq only affected CPT1A, not HK1.**

-We thank the reviewer for bringing this to our attention. We have now corrected this in the text and corresponding figure.

**7. In addition to profiling metabolic enzyme expression, the authors should conduct direct bioenergetic flux experiments to validate their MetFlow findings.**

-While we did not have enough matched pre- and post-PBMC samples remaining to conduct flux experiments such as Seahorse assays, we were able to profile metabolites in matched pre- and post-COVID patient serum. Metabolite signatures were distinct between pre- and post-COVID groups with particular depletion of TCA cycle intermediates. This is now shown in new figures 4A-C.

**8. Establish whether inhibitory/exhaustion signaling (PD‑1 axis) is mechanistically linked to the metabolic impairment.**

-While mechanistic experiments such as assessing pre- and post-COVID Ag-specific CTL killing of target cells would be ideal, we did not have enough matched samples or enough PBMCs to conduct this type of assay. We did, however, demonstrate an expansion of TCF1+PD1+ CD8 memory T cells (precursors of exhausted T cells, or T_pex_) in post-COVID samples (Figure S10E).

***Reviewer 3***

**1. The peptide pools used are described (IAV NA, VZV Orf4, S. aureus hemolysin A). It would be helpful to acknowledge the limitation that single-protein peptide pools may not fully represent pathogen-wide memory, especially for S. aureus.**

-We have acknowledged this in the limitations section (lines 516-518).

**2. The manuscript notes batch and age effects in top PCs. Please explicitly state how these were handled in differential expression/GSEA (e.g., covariate adjustment, batch correction) and provide sensitivity analyses when possible.**

-SCALES is a published method^1^, and we have also added more explanation in the results section (lines 195-205).

**3. Confirm that paired tests are consistently used for matched samples and specify when nonparametric tests were applied.**

-Figure legends now indicate that paired tests were used for all figures analyzing matched pre- and post-COVID samples. Parametric vs. nonparametric are also specified.

**5. Fig. 4H–I: Please specify the unit of analysis (donor-level correlation), the correlation metric (Spearman is noted), significance thresholds, and sample size contributing to each diagram to avoid any concern about pseudo-replication.**

-These parameters are specified in the legend for Figure 4.

**6. Provide vehicle controls, justification for doses and incubation times, and viability/toxicity checks to support interpretation of “rescue” effects.**

-Information on vehicls controls are in the results and figure legends. The doses and incubation times are taken from published studies looking at metformin/ubiquinol effects on T cell function and are cited in the paper. We also added new data showing no difference in PBMC cell viability after Met/Ubq exposure (Fig. S11).

***Minor issues***

**1. Fig S8 (PCR) text inconsistent with Ct table-undetermined.**

-We changed this to “below LoD” (limit of detection) in Fig. S12.

**2. In Figure 3F and I, there are no group indicators of green histograms. Similar issues are also in Figure 3G and J.**

-This has been corrected in Figure 3.

**3. It is confusing why the MFI values are negative. There is not description related to the data “norm” in the figure legends or method.**

-We have added a description of how we normalized MFI values in the legend of Figure 3.

**4. It supposed that the percentage numbers in Figure 3D, G and J are the proportions of samples, but it is not mentioned in the figure legend.**

-This has been appended in the figure legend.

**5. In Figure 4F, is the “-2.1” a typo? Also, the values are missing in the HK1 histogram.**

-These issues have been corrected in Figure 4.

**6. In Figure 4G, although the Y axis is cut to 0.6 or 0.7 in HK1 MFI and TKT MFI with statistical differences, the fold changes are very small between pre- and post-samples. It is unclear whether this tiny change can lead to biological difference.**

-We have added this qualification to the results (lines 295-296).

We thank the Editors and Reviewers for their valuable time and contributions in helping us improve our study. We appreciate that our revised manuscript is suitable for publication in *PLoS Pathogens*.

Sincerely,

Lavanya Visvabharathy, Ph.D

1.	Zaydman, M. A. *et al.* Defining hierarchical protein interaction networks from spectral analysis of bacterial proteomes. *eLife* **11**, e74104 (2022).
