# Supplementary material for: Post-COVID impairment of T cell responses to community-acquired pathogens can be modified by activating cellular metabolism
Source: PLoS Pathog. 2026 Jul 30;22(7):e1013860. doi: 10.1371/journal.ppat.1013860 (PMC13441075; doi:10.1371/journal.ppat.1013860)
Supplement: Supplementary file 18 [file ppat.1013860.s019.docx]

**Response to Reviewers’ Comments, Round 2**

We thank Reviewers 2 & 3 for recommending acceptance of our manuscript at *PLoS Pathogens*. We have now addressed all comments from Reviewer 4 and revised the text of the manuscript accordingly. The Reviewers’ comments appear **bolded** and are followed by our responses.

***Minor issues***

**1. The abstract is poorly written and requires improvement for better clarity and impact.**

We thank Reviewer 4 for this comment. We have edited the abstract to focus more closely on the important findings of our paper.

**2. What exclusion criteria were applied when selecting donors beyond being naïve or then having SARS CoV 2 infection? Were any immunocompromised individuals included in the study? Clarifying this information is important for interpreting the data. Please consider adding a paragraph in the Discussion to address this point (or in limitation section).**

We included naïve individuals who had prior exposure to IAV, SA, and VZV as determined by serology (Fig. S1). We now also include in the Limitations section that 2/31 patients were on immunomodulatory medications for autoimmune disease, though they had taken the medications for years prior to and after their first SARS-CoV-2 infection.

**3. The discussion should present the findings concisely. I recommend that the authors revise the discussion section accordingly.**

We thank Reviewer 4 for their comments. We agree that brevity is a strength; we revised the Discussion accordingly.

We thank the Editors and Reviewers for their valuable time and contributions in helping us improve our study. We hope that our revised manuscript is suitable for publication in *PLoS Pathogens*.

Sincerely,

Lavanya Visvabharathy, Ph.D
