# Supplementary material for: Post-COVID impairment of T cell responses to community-acquired pathogens can be modified by activating cellular metabolism
Source: PLoS Pathog. 2026 Jul 30;22(7):e1013860. doi: 10.1371/journal.ppat.1013860 (PMC13441075; doi:10.1371/journal.ppat.1013860)
Supplement: Peer review history

# Peer review: Decision Letter 0

**DOI:** 10.1371/journal.ppat.1013860.r001

**Authors:** Haitao Hu, Sonja Best

23 Feb 2026

PPATHOGENS-D-25-03310

Post-COVID impairment of memory T cell responses to community-acquired pathogens can be rectified by activating cellular metabolism

PLOS Pathogens

Dear Dr. Visvabharathy,

Thank you for submitting your manuscript to PLOS Pathogens. After careful consideration, we feel that it has merit but does not fully meet PLOS Pathogens's publication criteria as it currently stands. Therefore, we invite you to submit a revised version of the manuscript that addresses the points raised during the review process.

Please submit your revised manuscript by Apr 24 2026 11:59PM. If you will need more time than this to complete your revisions, please reply to this message or contact the journal office at plospathogens@plos.org. When you're ready to submit your revision, log on to https://www.editorialmanager.com/ppathogens/ and select the 'Submissions Needing Revision' folder to locate your manuscript file.

Please include the following items when submitting your revised manuscript:

* A letter that responds to each point raised by the editor and reviewer(s). You should upload this letter as a separate file labeled 'Response to Reviewers'. This file does not need to include responses to any formatting updates and technical items listed in the 'Journal Requirements' section below.

* A marked-up copy of your manuscript that highlights changes made to the original version. You should upload this as a separate file labeled 'Revised Manuscript with Track Changes'.

* An unmarked version of your revised paper without tracked changes. You should upload this as a separate file labeled 'Manuscript'.

If you would like to make changes to your financial disclosure, competing interests statement, or data availability statement, please make these updates within the submission form at the time of resubmission. Guidelines for resubmitting your figure files are available below the reviewer comments at the end of this letter.

We look forward to receiving your revised manuscript.

Kind regards,

Haitao Hu, PhD

Guest Editor

PLOS Pathogens

Sonja Best

Section Editor

PLOS Pathogens

Sumita Bhaduri-McIntosh

Editor-in-Chief

PLOS Pathogens

orcid.org/0000-0003-2946-9497

Michael Malim

Editor-in-Chief

PLOS Pathogens

orcid.org/0000-0002-7699-2064

**Additional Editor Comments:**

• The manuscript text notes that J.C.A. is the founder of Covira Pharmaceuticals (stated as unaffiliated), while other submission fields indicate no competing interests; this should be reconciled to ensure consistent disclosure.

• The statement that Boltzmann Brain analysis is proprietary and unavailable, which may be at odds with transparency expectations. The authors will provide: either (i) sufficient methodological and output disclosure to reproduce the key findings, or (ii) confirmation of key results via open, standard analytic approaches.

• Edit properly the section of “the population-level mortality/infection trend analysis” to avoid implying causality beyond what the cohort data can support

**Journal Requirements:**

If the reviewer comments include a recommendation to cite specific previously published works, please review and evaluate these publications to determine whether they are relevant and should be cited. There is no requirement to cite these works unless the editor has indicated otherwise.

1) Please ensure that the CRediT author contributions listed for every co-author are completed accurately and in full.

At this stage, the following Authors/Authors require contributions: Daniel D. Carroll, Kamacay Cira, Jack Archer, Jason Shapiro, Ue-Yu Pen, David Tieri, Lucia Leonor, Neda D. Roofchayee, Samantha S. Yee, Marc Wahab, Igor J. Koralnik, John C. Alverdy, Arjun S. Raman, and Lavanya Visvabharathy. Please ensure that the full contributions of each author are acknowledged in the "Add/Edit/Remove Authors" section of our submission form.

The list of CRediT author contributions may be found here: https://journals.plos.org/plospathogens/s/authorship#loc-author-contributions

2) We ask that a manuscript source file is provided at Revision. Please upload your manuscript file as a .doc, .docx, .rtf or .tex. If you are providing a .tex file, please upload it under the item type u2018LaTeX Source Fileu2019 and leave your .pdf version as the item type u2018Manuscriptu2019.

3) Please provide an Author Summary. This should appear in your manuscript between the Abstract (if applicable) and the Introduction, and should be 150-200 words long. The aim should be to make your findings accessible to a wide audience that includes both scientists and non-scientists. Sample summaries can be found on our website under Submission Guidelines:

https://journals.plos.org/plospathogens/s/submission-guidelines#loc-parts-of-a-submission

4) We noticed that you used the phrase 'data not shown' in the manuscript. We do not allow these references, as the PLOS data access policy requires that all data be either published with the manuscript or made available in a publicly accessible database. Please amend the supplementary material to include the referenced data or remove the references.

5) We do not publish any copyright or trademark symbols that usually accompany proprietary names, eg ©,  ®, or TM  (e.g. next to drug or reagent names). Therefore please remove all instances of trademark/copyright symbols throughout the text, including:

- TM on page: 19.

6) Please upload all main figures as separate Figure files in .tif or .eps format. For more information about how to convert and format your figure files please see our guidelines:

https://journals.plos.org/plospathogens/s/figures

7) We notice that your supplementary Figures are included in the manuscript file. Please remove them and upload them with the file type 'Supporting Information'. Please ensure that each Supporting Information file has a legend listed in the manuscript after the references list.

8) Some material included in your submission may be copyrighted. According to PLOSu2019s copyright policy, authors who use figures or other material (e.g., graphics, clipart, maps) from another author or copyright holder must demonstrate or obtain permission to publish this material under the Creative Commons Attribution 4.0 International (CC BY 4.0) License used by PLOS journals. Please closely review the details of PLOSu2019s copyright requirements here: PLOS Licenses and Copyright. If you need to request permissions from a copyright holder, you may use PLOS's Copyright Content Permission form.

Please respond directly to this email and provide any known details concerning your material's license terms and permissions required for reuse, even if you have not yet obtained copyright permissions or are unsure of your material's copyright compatibility. Once you have responded and addressed all other outstanding technical requirements, you may resubmit your manuscript within Editorial Manager.

Potential Copyright Issues:

i) Figures 1C, 2A, 4A, and 6. Please confirm whether you drew the images / clip-art within the figure panels by hand. If you did not draw the images, please provide (a) a link to the source of the images or icons and their license / terms of use; or (b) written permission from the copyright holder to publish the images or icons under our CC BY 4.0 license. Alternatively, you may replace the images with open source alternatives. See these open source resources you may use to replace images / clip-art:

- https://commons.wikimedia.org

- https://openclipart.org/.

**Reviewers' Comments:**

Reviewer's Responses to Questions

**Part I - Summary**

Please use this section to discuss strengths/weaknesses of study, novelty/significance, general execution and scholarship.

Reviewer #1: In this manuscript, the authors investigate the mechanism underlying the observed impairment of immune memory to community-acquired pathogens (such as VZV and Influenza) following SARS-CoV-2 infection. Using a longitudinal cohort of pre- and post-COVID samples, the study proposes a concept of "metabolic immobilization," suggesting that bystander memory T cells acquire a mitochondrial defect that prevents them from engaging the necessary metabolic programs (glycolysis and OXPHOS) upon antigen re-exposure. The authors employ metabolic flux analysis, bulk RNA-seq, and flow cytometry to characterize this dysfunction and provide ex vivo data suggesting that metabolic modulators could potentially restore these responses.

However, I have multiple concerns regarding the technical execution and interpretation of the data:

1) In the experimental design in Figure 2. The authors performed bulk RNA-seq on the total memory T cell after peptides stimulation, without sorting for antigen-specific AIM+ cells. Since VZV-specific T cells are typically rare (less than 0.1 shown in Figure 3B), the RNA-seq signal is likely dominated by non-specific bystander cells, which is a huge noise. Particularly, the Post-COVID samples contains expanded SARS-CoV-2 specific T cells, which are absent in the Pre-COVID controls. These COVID-specific clones could skew the metabolic profile. Therefore, the differences observed might simply reflect a change in the overall composition of the memory pool rather than a specific defect in VZV-reactive cells.

If no further data provided (for example, scRNAseq and/or TCRseq, or bulk RNAseq after AIM+ sorting), the authors should clearly acknowledge this limitation in the main text. Please also tone down the claims in the text, as the bulk RNA-seq results cannot definitively be attributed to "VZV-specific" reprogramming.

2) In Figure 3, the number of AIM+ events in many samples are very low (appears to be < 50 according to Figure S5). Calculating MFI on such rare populations is problematic. Low event counts make the mean/median highly susceptible to outliers. Particularly, mitochondrial dyes have high inherent staining heterogeneity. In the absence of a sufficient number of events to average out this variability, the MFI values likely reflect stochastic noise rather than true biological differences.

3) the authors observed an increase in mitochondrial metabolic markers in the bulk CD45RO+ population following peptides stimulation. This global upregulation suggests that the observed signal is driven most likely by bystander activation via paracrine signaling rather than direct TCR engagement of the majority population. Consequently, the reduced response seen in the Post-COVID group might reflect a generalized refractoriness to cytokine stimulation or a defect in bystander activation, rather than a specific impairment in VZV-specific TCR signaling or metabolic recall. The authors should distinguish between these two possibilities, as the current data does not strictly prove a defect in antigen-specific memory recall.

4) Regarding the interpretation of the "rescue" effects shown in Figure 5. The authors demonstrate that treatment restores mitochondrial parameters and cytokine production. However, Pharmacologically forcing these compromised mitochondria to increase OXPHOS activity could lead to excessive ROS production and accelerated apoptosis. I suspect that the cell viability or ROS levels may change after the treatment. Additionally, The treatment likely acts nonspecifically on the entire T cell pool. The observed increase in cytokine production might simply reflect a lowered activation threshold for the global population, rather than the restoration of the precise, antigen-specific memory program.

5) The concept of "Metabolic Immobilization" needs to be better contextualized within the existing literature on T cell exhaustion and immunosenescence. Post-COVID T cells show mitochondrial dysfunction and ROS accumulation, which are the features of both exhausted and senescent T cells. Is "Metabolic Immobilization" truly a distinct phenotype induced specifically by SARS-CoV-2, or is it simply a metabolic manifestation of generalized T cell exhaustion caused by systemic inflammation? Also, the authors should discuss whether this phenotype is unique to COVID-19 or likely a common sequela of any severe viral infection.

Reviewer #2: This manuscript leverages a valuable donor-matched, longitudinal design (pre- vs ~30 weeks post-COVID infection) in a cohort originally enrolled as COVID-naïve adults. The major conclusion is that post-COVID antigen-specific memory T cells exhibit metabolic immobilization, such as disordered mitochondrial and glycolytic programs, resulting in impaired responses to other pathogens. These dysregulated memory T cells can be partially rescued by metabolic agents. Therefore, this mechanism may contribute to elevated post-pandemic infection burden. The experimental platform (AIM-based antigen-specific gating, mitochondrial dyes, Met-Flow enzyme panels, and RNA-seq of stimulated CD4 memory cells) is appropriate. Overall, this study is of high significance and novelty; however, whether these results can support the central hypothesis is uncertain. Importantly, the T memory cell functional assay, such as cytokine production and cell proliferation under stimulation with peptides in pre- and post-CoV samples are not provided. In addition, some results are not described accurately, and certain information is missing from the figures and their legends.

Reviewer #3: This manuscript addresses an important and timely question: whether SARS CoV 2 infection leaves a durable imprint on adaptive immune recall to unrelated, commonly encountered pathogens through immunometabolic dysfunction. Using donor-matched pre and post COVID PBMC samples from a longitudinally followed cohort (n=31 total; multiple assays performed on subsets), the authors report: (i) transcriptional remodeling of VZV stimulated CD4 memory T cells with decreased activation/antigen-presentation programs but increased mitochondrial/OXPHOS-related pathways, (ii) impaired mitochondrial flux/ROS responses in antigen-stimulated, AIM+ memory T cells after COVID in a substantial fraction of donors, (iii) altered “connectivity” between activation markers and metabolic enzymes at the memory T-cell population level, and (iv) partial metabolic rescue of post COVID defects with metformin or ubiquinol.

Strengths

1.Major advantage for immunophenotyping studies and reduces inter-individual variability, strengthening inference about post infection changes

2.bulk RNA-seq (VZV-stimulated CD4 memory), functional mitochondrial dyes (TMRM/MitoSOX/Mitotracker), and metabolic enzyme profiling (Met Flow) provide convergent evidence for immunometabolic remodeling

3.inclusion of VZV, influenza A, and S. aureus supports the idea of a pathogen-independent post COVID effect on recall responses rather than an idiosyncratic antigen system.

4.Demonstrating partial reversal with two accessible agents (metformin/ubiquinol) increases potential clinical relevance and motivates future mechanistic and interventional work.

Weaknesses / limitations affecting conclusions

1.The central claim implies impaired recall (e.g., cytokine production, proliferative capacity, helper activity), yet the presented data are primarily metabolic surrogates (enzyme expression; mitochondrial dyes) and transcriptional shifts. Without direct functional readouts, it remains uncertain whether the observed metabolic remodeling translates into clinically meaningful impairment of recall responses.

2.Participants were enrolled during 2021–2024, were vaccinated with mRNA regimens, and acquired COVID at different times; post COVID sampling is reported as ~30 weeks after infection, but the range (3–9 months) and clinical severity, variant era, and booster timing may differ substantially. These covariates could influence both metabolism and activation markers and should be explicitly modeled/stratified.

3.The RNA-seq uses SCALES and the flow “Boltzmann Brain” embedding is described as proprietary and not shareable, limiting independent validation and potentially raising editorial concerns about transparency. At minimum, the paper needs to ensure the main conclusions are robust to standard, open methods.

4.The CDC WONDER analyses (Fig. 1) are ecological and could be influenced by many post-pandemic factors (healthcare utilization patterns, coding practices, antibiotic resistance dynamics, etc.). The manuscript currently risks implying a causal link from the cohort-based immunometabolic findings to national mortality trends, which is not directly tested.

5.Supplementary Fig. S8 text appears inconsistent with the Ct table (“Undetermined” for N1/N2 in samples), and the legend wording is confusing.

**********

**Part II – Major Issues: Key Experiments Required for Acceptance**

Please use this section to detail the key new experiments or modifications of existing experiments that should be absolutely required to validate study conclusions.

Generally, there should be no more than 3 such required experiments or major modifications for a "Major Revision" recommendation. If more than 3 experiments are necessary to validate the study conclusions, then you are encouraged to recommend "Reject".

Reviewer #1: (No Response)

Reviewer #2: 1. The increased infection rate after COVID-19 pandemic is observed, but the reasons are complicate. For example, the routine vaccination disruption due to the alternated people recognition may leave immunity gaps that later fueled outbreaks (e.g., measles, pertussis). Bacterial diseases often rise after viral waves because viral infections can damage mucosa and alter immune defenses. Some animal experiments suggest that this altered immunity by viral infection, such as impaired pulmonary innate immune response (e.g., AM, IFN-I) is the key mechanism resulting in subsequently increased bacterial infection (PMID: 26001778, 19487810, 39879546). However, the extent to which dysfunctional memory T cells drive the observed increase in infection rates in humans remains unclear. Although human data is very critical and valuable, more evidence from animal experiments might be needed in future to determine the role of T memory cell impairment and their contribution to other infection.

2. The cohort was recruited under a COVID vaccination study, and subjects were vaccinated with Pfizer/Moderna during the study. The information including vaccination timing, disease severity, treatments, comorbidities, and time-since-infection distribution during the 30 weeks needs to be clarified.

3. The impairment of memory T cell response in this study is largely inferred from metabolic coordination/markers rather than direct recall function (e.g., cytokine output, proliferation, cytotoxicity). The analysis of antigen-stimulated cytokine readouts (intracellular cytokines or secreted IFN-γ/IL-2/TNF), proliferation (CFSE labeling), or degranulation (CD107a) under the stimulation of peptides will strengthen the conclusion.

4. In line It is unclear why COVID, but not HCV or influenza, is capable of inducing downregulated glycolytic proteins in T cells. What is the underlying mechanism by which COVID dysregulates mitochondrial function in memory T cells? The author needs to discuss this.

5. In lines 294-296, the author mentioned that metformin or ubiquinol had a demonstrable effect in enhancing HK1 and CPT1A expression, however, the column data did not show any differences between post-Cov and pos-Cov/Met or pos-Cov/Ubq for HK1% and HK1 MFI.

Reviewer #3: 1) Demonstrate that post COVID antigen-specific memory T cells have impaired functional recall (not only metabolic signatures)

2) Validate the metabolic “immobilization” using direct bioenergetic flux measurements in relevant populations

3) Establish whether inhibitory/exhaustion signaling (PD‑1 axis) is mechanistically linked to the metabolic impairment

**********

**Part III – Minor Issues: Editorial and Data Presentation Modifications**

Please use this section for editorial suggestions as well as relatively minor modifications of existing data that would enhance clarity.

Reviewer #1: (No Response)

Reviewer #2: 1. In Figure 3F and I, there are no group indicators of green histograms. Similar issues are also in Figure 3G and J. In addition, it is confusing why the MFI values are negative. There is not description related to the data “norm” in the figure legends or method. It supposed that the percentage numbers in Figure 3D, G and J are the proportions of samples, but it is not mentioned in the figure legend. Both MFI and gMFI are presented in this figure, which makes it unclear whether they represent the same or different statistical measures.

2. In Figure 4F, is the “-2.1” a typo? Also, the values are missing in the HK1 histogram. In Figure 4G, although the Y axis is cut to 0.6 or 0.7 in HK1 MFI and TKT MFI with statistical differences, the fold changes are very small between pre- and post-samples. It is unclear whether this tiny change can lead to biological difference.

Reviewer #3: 1. The CDC WONDER analysis can remain as rationale, but discussion should clearly distinguish ecological associations from mechanistic inference in this cohort.

2. Please report (in Results or a dedicated table): time from infection to post sample (median, IQR/range), COVID severity (including hospitalization/antivirals if applicable), vaccination/booster timing relative to sampling, and criteria for any “long COVID” categorization/exclusion (the discussion states findings in those without long COVID).

3. The peptide pools used are described (IAV NA, VZV Orf4, S. aureus hemolysin A). It would be helpful to acknowledge the limitation that single-protein peptide pools may not fully represent pathogen-wide memory, especially for S. aureus.

4. The manuscript notes batch and age effects in top PCs. Please explicitly state how these were handled in differential expression/GSEA (e.g., covariate adjustment, batch correction) and provide sensitivity analyses when possible.

5. Because Boltzmann Brain is stated to be proprietary/unavailable, please ensure the main conclusions (e.g., PD 1/GLUT1/HLA DR shifts; connectivity differences) are supported by standard, open analyses (e.g., regression models, PCA/UMAP with clear feature importances), or provide enough methodological detail and derived outputs to allow independent verification.

6. Confirm that paired tests are consistently used for matched samples and specify when nonparametric tests were applied. Consider reporting effect sizes and confidence intervals to complement p-values.

7. Supplementary Fig. S8 : The Ct table shows “Undetermined” for N1/N2 in samples while positive/negative controls are listed. The figure legend text should be corrected for consistency and clarity.

8. Fig. 3: If bimodality is emphasized, consider adding a simple quantitative approach (e.g., threshold-based classification with rationale) and provide donor counts by antigen and endpoint (flux/ROS).

9. Fig. 4H–I: Please specify the unit of analysis (donor-level correlation), the correlation metric (Spearman is noted), significance thresholds, and sample size contributing to each diagram to avoid any concern about pseudo-replication.

10. Provide vehicle controls, justification for doses and incubation times, and viability/toxicity checks to support interpretation of “rescue” effects.

11. Address minor typographical/format inconsistencies (e.g., spacing in “Varicella zoster,” double punctuation, PD 1 vs PD1 formatting) and ensure AIM+ gating definitions are clearly and consistently stated in Results.

**********

PLOS authors have the option to publish the peer review history of their article (what does this mean?). If published, this will include your full peer review and any attached files.

If you choose “no”, your identity will remain anonymous but your review may still be made public.

**Do you want your identity to be public for this peer review?**  For information about this choice, including consent withdrawal, please see our Privacy Policy.

Reviewer #1: No

Reviewer #2: No

Reviewer #3: No

**Figure resubmission:**

-->While revising your submission, we strongly recommend that you use PLOS’s NAAS tool (https://ngplosjournals.pagemajik.ai/artanalysis) to test your figure files. NAAS can convert your figure files to the TIFF file type and meet basic requirements (such as print size, resolution), or provide you with a report on issues that do not meet our requirements and that NAAS cannot fix.

After uploading your figures to PLOS’s NAAS tool - https://ngplosjournals.pagemajik.ai/artanalysis, NAAS will process the files provided and display the results in the "Uploaded Files" section of the page as the processing is complete. If the uploaded figures meet our requirements (or NAAS is able to fix the files to meet our requirements), the figure will be marked as "fixed" above. If NAAS is unable to fix the files, a red "failed" label will appear above. When NAAS has confirmed that the figure files meet our requirements, please download the file via the download option, and include these NAAS processed figure files when submitting your revised manuscript.

**Reproducibility:**

To enhance the reproducibility of your results, we recommend that authors of applicable studies deposit laboratory protocols in protocols.io, where a protocol can be assigned its own identifier (DOI) such that it can be cited independently in the future. Additionally, PLOS ONE offers an option to publish peer-reviewed clinical study protocols. Read more information on sharing protocols at https://plos.org/protocols?utm_medium=editorial-email&utm_source=authorletters&utm_campaign=protocols

# Author comment: Author response to Decision Letter 1

**DOI:** 10.1371/journal.ppat.1013860.r002

3 Jun 2026

Attachment Submitted filename: Response to Reviewers-PlosPath.docx

# Peer review: Decision Letter 1

**DOI:** 10.1371/journal.ppat.1013860.r003

**Authors:** Haitao Hu, Sonja Best

10 Jul 2026

PPATHOGENS-D-25-03310R1

Post-COVID impairment of T cell responses to community-acquired pathogens can be modulated by activating cellular metabolism

PLOS Pathogens

Dear Dr. Lavanya Visvabharathy,

Thank you for submitting your manuscript to PLOS Pathogens. After careful consideration, we feel that it has merit but does not fully meet PLOS Pathogens's publication criteria as it currently stands. Therefore, we invite you to submit a revised version of the manuscript that addresses the points raised during the review process, especially those from Reviewer 4.

Please submit your revised manuscript by Sep 08 2026 11:59PM. If you will need more time than this to complete your revisions, please reply to this message or contact the journal office at plospathogens@plos.org. When you're ready to submit your revision, log on to https://www.editorialmanager.com/ppathogens/ and select the 'Submissions Needing Revision' folder to locate your manuscript file.

Please include the following items when submitting your revised manuscript:

* A letter that responds to each point raised by the editor and reviewer(s). You should upload this letter as a separate file labeled 'Response to Reviewers'. This file does not need to include responses to any formatting updates and technical items listed in the 'Journal Requirements' section below.

* A marked-up copy of your manuscript that highlights changes made to the original version. You should upload this as a separate file labeled 'Revised Manuscript with Track Changes'.

* An unmarked version of your revised paper without tracked changes. You should upload this as a separate file labeled 'Manuscript'.

If you would like to make changes to your financial disclosure, competing interests statement, or data availability statement, please make these updates within the submission form at the time of resubmission. Guidelines for resubmitting your figure files are available below the reviewer comments at the end of this letter.

As the corresponding author, your ORCID iD is verified in the submission system and will appear in the published article. PLOS supports the use of ORCID, and we encourage all coauthors to register for an ORCID iD and use it as well. Please encourage your coauthors to verify their ORCID iD within the submission system before final acceptance, as unverified ORCID iDs will not appear in the published article. Only the individual author can complete the verification step; PLOS staff cannot verify ORCID iDs on behalf of authors.

We look forward to receiving your revised manuscript.

Kind regards,

Haitao Hu, PhD

Guest Editor

PLOS Pathogens

Sonja Best

Section Editor

PLOS Pathogens

Sumita Bhaduri-McIntosh

Editor-in-Chief

PLOS Pathogens

orcid.org/0000-0003-2946-9497

Michael Malim

Editor-in-Chief

PLOS Pathogens

orcid.org/0000-0002-7699-2064

**Additional Editor Comments (if provided):**

**Journal Requirements:**

If the reviewer comments include a recommendation to cite specific previously published works, please review and evaluate these publications to determine whether they are relevant and should be cited. There is no requirement to cite these works unless the editor has indicated otherwise.

1) We have noticed that you have uploaded Supporting Information files (Tables) , but you have not included a list of legends. Please add a full list of legends for your Supporting Information files after the references list.

**Reviewers' Comments:**

Reviewer's Responses to Questions

**Part I - Summary**

Please use this section to discuss strengths/weaknesses of study, novelty/significance, general execution and scholarship.

Reviewer #2: This manuscript has been well revised and is suitable for consideration for acceptance.

Reviewer #3: The authors have adequately addressed the concerns raised in the previous review. The revised manuscript has been substantially improved.

Reviewer #4: Strengths:

- The data are clearly presented and strongly support the central hypothesis.

- The authors employed multiple complementary techniques to generate and validate the findings.

- RNA‑seq results were corroborated using conventional molecular methods, increasing confidence in the conclusions.

- The study’s findings have substantial impact and advance understanding in the field.

Weaknesses:

- The abstract and discussion sections are poorly written and require substantial revision to improve clarity and coherence.

- The samples used in this study lack documented histories of prior viral infections, which limits the interpretation of the immune signatures observed.

**********

**Part II – Major Issues: Key Experiments Required for Acceptance**

Please use this section to detail the key new experiments or modifications of existing experiments that should be absolutely required to validate study conclusions.

Generally, there should be no more than 3 such required experiments or major modifications for a "Major Revision" recommendation. If more than 3 experiments are necessary to validate the study conclusions, then you are encouraged to recommend "Reject".

Reviewer #2: All issues have been addressed.

Reviewer #3: none

Reviewer #4: No major issue was observed

**********

**Part III – Minor Issues: Editorial and Data Presentation Modifications**

Please use this section for editorial suggestions as well as relatively minor modifications of existing data that would enhance clarity.

Reviewer #2: All issues have been addressed.

Reviewer #3: none

Reviewer #4: Minor:

- The abstract is poorly written and requires improvement for better clarity and impact.

- What exclusion criteria were applied when selecting donors beyond being naïve or then having SARS CoV 2 infection? Were any immunocompromised individuals included in the study? Clarifying this information is important for interpreting the data. Please consider adding a paragraph in the Discussion to address this point (or in limitation section).

- The discussion should present the findings concisely. I recommend that the authors revise the discussion section accordingly.

**********

PLOS authors have the option to publish the peer review history of their article (what does this mean?). If published, this will include your full peer review and any attached files.

If you choose “no”, your identity will remain anonymous but your review may still be made public.

**Do you want your identity to be public for this peer review?**  For information about this choice, including consent withdrawal, please see our Privacy Policy.

Reviewer #2: No

Reviewer #3: No

Reviewer #4: No

[NOTE: If reviewer comments were submitted as an attachment file, they will be attached to this email and accessible via the submission site. Please log into your account, locate the manuscript record, and check for the action link "View Attachments". If this link does not appear, there are no attachment files.]

**Figure resubmission:**

-->While revising your submission, we strongly recommend that you use PLOS’s NAAS tool (https://ngplosjournals.pagemajik.ai/artanalysis) to test your figure files. NAAS can convert your figure files to the TIFF file type and meet basic requirements (such as print size, resolution), or provide you with a report on issues that do not meet our requirements and that NAAS cannot fix.

After uploading your figures to PLOS’s NAAS tool - https://ngplosjournals.pagemajik.ai/artanalysis, NAAS will process the files provided and display the results in the "Uploaded Files" section of the page as the processing is complete. If the uploaded figures meet our requirements (or NAAS is able to fix the files to meet our requirements), the figure will be marked as "fixed" above. If NAAS is unable to fix the files, a red "failed" label will appear above. When NAAS has confirmed that the figure files meet our requirements, please download the file via the download option, and include these NAAS processed figure files when submitting your revised manuscript.

**Reproducibility:**

To enhance the reproducibility of your results, we recommend that authors of applicable studies deposit laboratory protocols in protocols.io, where a protocol can be assigned its own identifier (DOI) such that it can be cited independently in the future. Additionally, PLOS ONE offers an option to publish peer-reviewed clinical study protocols. Read more information on sharing protocols at https://plos.org/protocols?utm_medium=editorial-email&utm_source=authorletters&utm_campaign=protocols

Attachment Submitted filename: Reviewer comments_PPATHOGENS-D-25-03310_R1.pdf

# Author comment: Author response to Decision Letter 2

**DOI:** 10.1371/journal.ppat.1013860.r004

13 Jul 2026

Attachment Submitted filename: Response to Reviewers_2.docx

# Editor report: Decision Letter 2

**DOI:** 10.1371/journal.ppat.1013860.r005

**Authors:** Haitao Hu, Sonja Best

16 Jul 2026

Dear Dr. Lavanya Visvabharathy,

We are pleased to inform you that your manuscript 'Post-COVID impairment of T cell responses to community-acquired pathogens can be modulated by activating cellular metabolism' has been provisionally accepted for publication in PLOS Pathogens.

Before your manuscript can be formally accepted you will need to complete some formatting changes, which you will receive in a follow up email. A member of our team will be in touch with a set of requests.

Please note that your manuscript will not be scheduled for publication until you have made the required changes, so a swift response is appreciated.

IMPORTANT: The editorial review process is now complete. PLOS will only permit corrections to spelling, formatting or significant scientific errors from this point onwards. Requests for major changes, or any which affect the scientific understanding of your work, will cause delays to the publication date of your manuscript.

Should you, your institution's press office or the journal office choose to press release your paper, you will automatically be opted out of early publication. We ask that you notify us now if you or your institution is planning to press release the article. All press must be co-ordinated with PLOS.

Thank you again for supporting Open Access publishing; we are looking forward to publishing your work in PLOS Pathogens.

Best regards,

Haitao Hu, PhD

Guest Editor

PLOS Pathogens

Sonja Best

Section Editor

PLOS Pathogens

Sumita Bhaduri-McIntosh

Editor-in-Chief

PLOS Pathogens

orcid.org/0000-0003-2946-9497

Michael Malim

Editor-in-Chief

PLOS Pathogens

orcid.org/0000-0002-7699-2064

***********************************************************

Reviewer Comments (if any, and for reference):

# Editor report: Acceptance letter

**DOI:** 10.1371/journal.ppat.1013860.r006

**Authors:** Haitao Hu, Sonja Best

Dear Dr. Visvabharathy,

We are delighted to inform you that your manuscript, "Post-COVID impairment of T cell responses to community-acquired pathogens can be modulated by activating cellular metabolism," has been formally accepted for publication in PLOS Pathogens.

We have now passed your article onto the PLOS Production Department who will complete the rest of the pre-publication process. All authors will receive a confirmation email upon publication.

The corresponding author will soon be receiving a typeset proof for review, to ensure errors have not been introduced during production. Please review the PDF proof of your manuscript carefully, as this is the last chance to correct any scientific or type-setting errors. Please note that major changes, or those which affect the scientific understanding of the work, will likely cause delays to the publication date of your manuscript. Note: Proofs for Front Matter articles (Pearls, Reviews, Opinions, etc...) are generated on a different schedule and may not be made available as quickly.

Soon after your final files are uploaded, the early version of your manuscript, if you opted to have an early version of your article, will be published online. The date of the early version will be your article's publication date. The final article will be published to the same URL, and all versions of the paper will be accessible to readers.

For Research Articles, you will receive an invoice from PLOS for your publication fee after your manuscript has reached the completed accept phase. If you receive an email requesting payment before acceptance or for any other service, this may be a phishing scheme. Learn how to identify phishing emails and protect your accounts at https://explore.plos.org/phishing.

Thank you again for supporting open-access publishing; we are looking forward to publishing your work in PLOS Pathogens.

Best regards,

Sumita Bhaduri-McIntosh

Editor-in-Chief

PLOS Pathogens

orcid.org/0000-0003-2946-9497

Michael Malim

Editor-in-Chief

PLOS Pathogens

orcid.org/0000-0002-7699-2064
